# Macrophages: Subtypes, Distribution, Polarization, Immunomodulatory Functions, and Therapeutics

**DOI:** 10.1002/mco2.70304

**Published:** 2025-07-25

**Authors:** Mengyuan Peng, Niannian Li, Hongbo Wang, Yaxu Li, Hui Liu, Yanhua Luo, Bao Lang, Weihang Zhang, Shilong Li, Liujun Tian, Bin Liu

**Affiliations:** ^1^ School of Anesthesiology Shandong Second Medical University Weifang China; ^2^ The First Affiliated Hospital of Shandong Second Medical University Weifang China

**Keywords:** inflammation, immune, macrophages, polarization, therapeutics, tissue‐resident macrophages

## Abstract

Macrophages are heterogeneous immune cells with diverse subtypes and tissue‐specific distributions, displaying dynamic polarization states that critically govern their immunomodulatory functions and responses to environmental cues. As key regulators of innate and adaptive immunity, they originate from either embryonic progenitors or bone marrow‐derived monocytes and exhibit remarkable plasticity in response to microenvironmental cues. Tissue‐resident macrophages (e.g., Langerhans cells, Kupffer cells, microglia) display unique organ‐specific functions, while inflammatory stimuli drive their polarization into proinflammatory (M1) or anti‐inflammatory (M2) phenotypes along a functional continuum. This review systematically examines macrophage subtypes, their anatomical distribution, and the signaling pathways (e.g., NF‐κB, STATs, PPARγ) underlying polarization shifts in acute and chronic inflammation. We highlight how polarization imbalances contribute to pathologies including neuroinflammation, liver fibrosis, and impaired tissue repair, particularly in aging contexts. Furthermore, we discuss emerging therapeutic strategies targeting macrophage plasticity, such as cytokine modulation, metabolic reprogramming, and subtype‐specific interventions. By integrating recent advances in macrophage biology, this work provides a comprehensive framework for understanding their dual roles in immune regulation and tissue homeostasis, offering insights for treating inflammatory and age‐related diseases through macrophage‐centered immunomodulation.

## Graphical abstract

Macrophages originate from the yolk sac, fetal liver, and bone marrow, differentiating into two main subtypes: M1‐like (proinflammatory) and M2‐like (anti‐inflammatory). These subtypes exhibit high plasticity, allowing them to transform in response to environmental cues or therapeutic interventions. In various chronic inflammatory conditions, such as aging and cancer, the polarization and functions of macrophages in various tissues (including liver, lung, kidney, skin, and brain) undergo significant alterations. They enter an imbalanced state, characterized by excessive M1‐like activation, weakened phagocytosis, aggravated inflammation, and muscle atrophy. Senescence‐associated secretory phenotype (SASP) factors further exacerbate these changes, leading to chronic inflammation and tissue dysfunction.

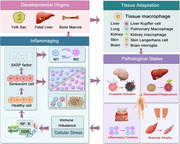

## 1 Introduction

Macrophages, first identified by Ilya Metchnikoff in the late 19th century as phagocytic defenders, are now recognized as central orchestrators of immunity, tissue homeostasis, and disease pathogenesis [1, 2]. Recent single‐cell technologies have revolutionized our understanding of macrophage biology, revealing an unexpected diversity in their developmental origins, tissue‐specific identities, and functional states. While traditionally viewed as terminally differentiated descendants of circulating monocytes [3, 4, 5], we now know many tissue‐resident populations originate from embryonic precursors and maintain themselves through local self‐renewal. This paradigm shift has profound implications for understanding their roles in health and disease [6].

The urgency to comprehensively review macrophage biology stems from three critical gaps in current knowledge. First, despite growing appreciation of macrophage heterogeneity, there remains a pressing need to systematically integrate findings across different tissues and pathological contexts. Second, the molecular mechanisms governing macrophage polarization—particularly during aging—remain incompletely understood [7, 8, 9, 10], even as global population aging makes this question increasingly clinically relevant. Third, while macrophage‐targeted therapies hold tremendous therapeutic promise, translating mechanistic insights into clinical applications continues to present significant challenges. This review aims to bridge these gaps by synthesizing cutting‐edge research with a particular focus on therapeutic implications.

Our work offers several key advances over previous reviews. We provide the first comprehensive analysis comparing macrophage subtypes across major organ systems, emphasizing how developmental origin dictates functional specialization. The review introduces a unified framework for understanding polarization dynamics along the M1–M2 spectrum [11, 12, 13, 14], incorporating groundbreaking discoveries about metabolic regulation and epigenetic control. Notably, we dedicate special attention to how aging reshapes macrophage biology, contributing to the phenomenon of “inflammaging” and age‐related pathologies [7, 8, 9, 10, 15, 16, 17, 18]. The therapeutic strategies section moves beyond conventional approaches to discuss emerging techniques like single‐cell‐guided interventions and nanotechnology‐based targeting.

This review examines macrophage biology through five integrated sections. We begin by establishing the developmental and anatomical basis of macrophage diversity (Section 2), then delve into the molecular mechanisms controlling polarization (Section 3). Section 4 provides a detailed analysis of tissue‐specific manifestations in skin, brain, liver, and muscle microenvironments, while Section 5 translates these fundamental insights into innovative therapeutic strategies. One of the unique features of our work is that we always emphasize the differences in the biological states and functions of macrophages between healthy and diseased conditions, ranging from the basic mechanisms to the clinical significance. Through this structure, we aim to provide both a fundamental resource for researchers and a practical guide for clinicians working at the intersection of immunology, aging biology, and precision medicine.

## 2 Three Origins of Macrophages

The characteristics and functions of macrophages are jointly determined by their developmental origins and tissue microenvironments. Embryonic‐derived macrophages (originating from yolk sac [YS] progenitors and fetal liver [FL]) establish long‐term resident tissue networks through self‐renewal, primarily maintaining homeostasis. In contrast, bone marrow (BM)‐derived monocytes differentiate via hematopoietic programs into either inflammatory or reparative subsets, dynamically adapting to microenvironmental demands. This section examines the functional consequences of this developmental dichotomy: in steady‐state conditions, embryonically derived macrophages (e.g., microglia and Kupffer cells) mediate immune surveillance, whereas BM‐derived monocytes preferentially repopulate compartments like the gut and skin. Furthermore, we highlight how developmental origins regulate inflammatory polarization dynamics—specifically, the temporal transition from proinflammatory (M1) to reparative (M2) states—and explore mechanisms underlying their dysregulation during pathological conditions.

### 2.1 Developmental Origins and Functional Diversity of Tissue Macrophages

**FIGURE 1:**
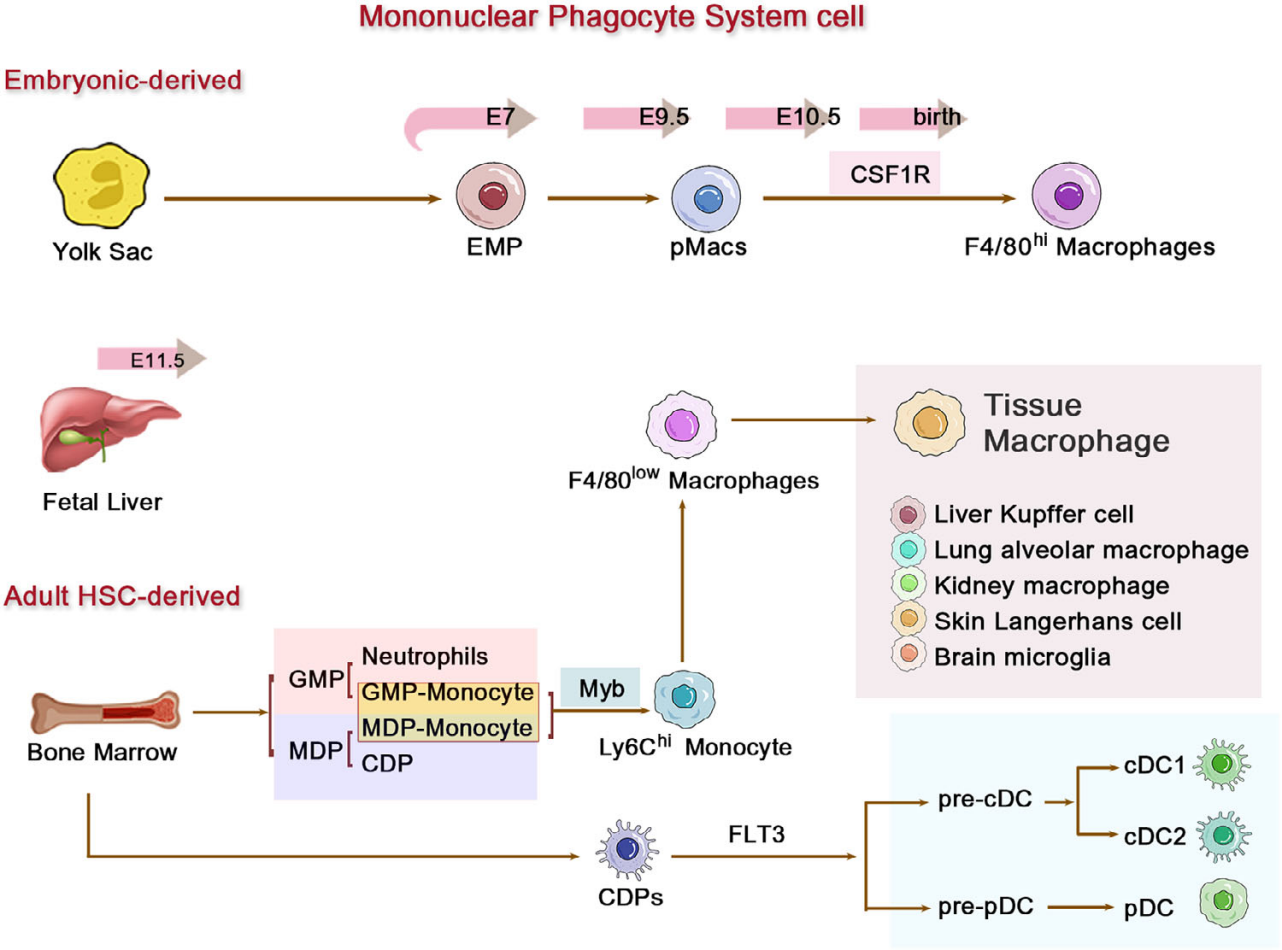
Developmental origins and heterogeneity of macrophages in the mononuclear phagocyte system. Macrophages from the three distinct origins exhibit tissue‐specific adaptation in different organs. The arrows in the figure indicate differentiation trajectories, highlighting the developmental differences between self‐renewing embryonic macrophages and dynamically replenished monocyte‐derived populations, along with their associated specific markers.

Emerging insights from recent lineage‐tracing studies have revolutionized our understanding of macrophage ontogeny, revealing at least three sources: primitive YS progenitors, FL monocytes, and BM monocytes [19]. The former two (YS and FL) are collectively termed embryonically derived macrophages, which are characterized by high expression of CX3CR1 and low expression of CCR2 and are hence referred to as CCR2‐negative resident macrophages (Figure 1). The first wave of hematopoietic progenitors appears in the extraembryonic YS, leading to primitive hematopoiesis, between embryonic days 7 and 9 (E7–E9) [20], generating CX3CR1‐dependent tissue‐resident macrophages that seed organs by E9.5. These F4/80^high^ macrophages maintaining tissues through Myb‐independent, colony‐stimulating factor 1 receptor (CSF‐1R)‐regulated self‐renewal [21, 22]. The second wave originates from FL monocytes (post‐E11.5), supplementing non‐central nervous system (CNS) tissues. Both YS‐ and FL‐derived populations exhibit longevity and self‐renewal capacity, forming the foundational tissue‐resident macrophage network that mediates immune surveillance, pathogen clearance, and tissue homeostasis throughout life [19, 23, 24, 25]. In the late stages of development, as newborn bones form, the hematopoietic function of the FL diminishes, and the BM assumes the primary responsibility for continuous hematopoiesis throughout the organism's life, occurring in an Myb‐dependent manner for all hematopoietic stem cells (HSCs) lineages and persisting into adulthood [19].

Monocytes originate from Ly6c^high^ monocytes in the BM, mature, and then enter the bloodstream and tissues. In mice, monocytes can be broadly categorized based on the expression levels of CCR2, Ly6C, and CX3CR1 [26, 27]: classical or proinflammatory monocytes expressing high levels of CCR2 and Ly6C and low levels of CX3CR1 (CCR2^+^Ly6C^hi^CX3CR1^low^, corresponding to CD14^++^CD16^−^ in humans); nonclassical, patrolling, or alternative monocytes expressing low levels of CCR2 and Ly6C and high levels of CX3CR1 (CCR2^−^Ly6C^low^CX3CR1^hi^, corresponding to CD14^+^CD16^++^ in humans); and intermediate monocytes expressing intermediate levels of Ly6C (Ly6C^int^, corresponding to CD14^++^CD16^+^ in humans) [26, 28, 29, 30].

### 2.2 The Dual Pathways of Differentiation Driven by Microenvironmental Signals

The developmental origins of tissue macrophages reveal a remarkable dichotomy that underpins their functional specialization. While YS‐derived macrophages establish long‐lived, self‐renewing populations in many tissues, HSC‐derived monocytes contribute to more dynamic macrophage compartments. This dual‐origin system creates distinct functional hierarchies across organs: alveolar macrophages and most cardiac/peritoneal macrophages predominantly originate from YS progenitors, maintaining stable populations through local proliferation [31]. Microglia represent the most extreme example of this developmental paradigm, maintaining their YS‐derived lineage throughout life with minimal monocyte contribution—a unique adaptation to the immune‐privileged CNS environment [32, 33, 34]. In striking contrast, macrophage populations in the intestinal mucosa, spleen, and dermis undergo continuous replenishment from circulating monocytes, reflecting the need for rapid adaptation to environmental challenges [22, 31, 35].

Even within organs containing both lineages, this developmental dichotomy creates functional specialization. The skin exemplifies this principle through its Langerhans cells: the prenatal YS‐derived population (established at E9.5–E10.0 in mice) forms a stable network capable of autonomous self‐renewal [23, 24, 25], while a separate monocyte‐derived subset undergoes rapid turnover (approximately 10 days) and mediates wound healing responses [1, 36, 37]. Although developmentally related to macrophages, these cells functionally resemble dendritic cells (DCs) through their unique capacity to recruit neutrophils via chemokine secretion at postcapillary venules [38, 39]. The liver presents an equally compelling case of developmental mosaicism. Kupffer cells, the liver's resident macrophages, originate primarily through primitive hematopoiesis before HSC emergence, but receive ongoing contributions from adult monocytes [38, 40]. Macrophage CSF (M‐CSF, CSF‐1) serves as the master regulator of this equilibrium—its deficiency impairs Kupffer cell maturation, while supplementation expands the population. Intriguingly, these developmentally distinct subsets (embryonic vs. monocyte‐derived) exhibit divergent gene expression profiles (e.g., CD163/CCR3 differences [38]) but converge functionally during inflammation, jointly participating in migratory and signaling responses [25, 41]. This stands in stark contrast to microglia, which remain developmentally isolated from circulating monocytes throughout the lifespan—a specialization that makes them unique within the mononuclear phagocyte system [32, 42, 43, 44].

Macrophage functional diversity stems not only from tissue‐specific adaptations but also from intrinsic heterogeneity in their monocyte precursors. While tissue‐resident macrophages of embryonic origin exhibit stable, self‐renewing properties, the circulating monocyte compartment serves as a dynamic reservoir capable of generating specialized effector populations in response to homeostatic or inflammatory cues. Classical monocytes (CCR2^+^Ly6C^hi^ in mice, CD14^+^CD16^−^ in humans) represent the primary source of tissue‐infiltrating macrophages, demonstrating remarkable plasticity in both steady‐state maintenance and inflammatory responses [30, 45].

Recent advances in hematopoietic lineage tracing have revealed that this functional versatility stems from two distinct BM differentiation pathways: 1, granulocyte–monocyte progenitors (GMP) generate “neutrophil‐like” inflammatory monocytes equipped for extracellular trap formation and rapid recruitment, yet limited in antigen‐presenting capacity. 2, monocyte‐DC progenitors (MDP) give rise to Ly6C^hi^ monocytes with DC potential, but lack granulocyte‐producing ability [46, 47]. This bifurcation in monocyte ontogeny enables precise immune adaptation—GMP‐derived monocytes dominate acute bacterial infections through neutrophil‐mimetic functions, while MDP‐lineage cells preferentially respond to viral challenges via DC‐like antigen presentation. Crucially, the BM dynamically adjusts the output of these progenitor pools based on systemic demands, with GMP expansion during sterile inflammation and MDP amplification in chronic infections [46, 47]. The tissue‐specific fates of these monocyte descendants are preprogrammed by their hematopoietic origins [16]. As detailed earlier, the same HSC‐derived monocytes can differentiate into: proinflammatory effectors in the inflamed dermis, tolerogenic macrophages in the steady‐state gut, or angiogenic Kupffer cells in regenerating liver. This developmental “priming” ensures that circulating monocytes not only replenish tissue macrophage pools, but do so in a manner tailored to each organ's physiological requirements—a paradigm we will explore further in the context of polarization dynamics.

### 2.3 Functional Polarization Dynamics in Inflammatory Responses Associated with Origin

Following their developmental specification (Sections 2.1 and 2.2), macrophages exhibit functional plasticity during inflammation. Macrophages are broadly categorized as resident tissue macrophages or inflammatory infiltrating subsets based on functional states. Under homeostatic conditions, resident macrophages primarily derive from the YS, independent of circulating monocytes and HSC, allowing for local self‐renewal, and exhibiting longer lifespans. However, during pathological stimulation or steady‐state inflammatory responses, BM‐derived inflammatory monocytes are recruited in a Myb‐dependent manner. Upon migrating to lymph nodes or injured tissues, these monocytes differentiate into F4/80^low^ macrophages regulated by FLT3, with a lifespan typically lasting only 1–2 days [26, 48]. During acute injury or inflammation, various inflammatory factors released from damaged and necrotic cells activate pattern recognition receptors on macrophages. Activated platelets and tissue‐resident macrophages stimulate the production of proinflammatory chemokines (e.g., CXCL‐1, CXCL‐2, CXCL‐5, CXCL‐8, and CX3CL1) and cytokines (e.g., TNF, IFN‐γ, IL‐1β, IL‐6, IL‐33, and growth factors [GFs]) [49, 50]. Proinflammatory chemokines, particularly CCL2 [51, 52], along with lipopolysaccharide (LPS), initiate the recruitment and activation of other immune cells, such as two subsets of monocytes, comprising CCR2^+^Ly6C^hi^ (human CD14^+^CD16^Neg^) and CX3CR1^+^Ly6C^low^ (human CD14^Neg^CD16^+^), which migrate from the adult BM to the injury site [50, 53, 54]. Once CCR2^+^Ly6C^hi^ monocytes extravasate into the interstitial tissue, they can differentiate into classically activated macrophages with proinflammatory phenotypes [55]. During the inflammatory phase, neutrophils and M1‐like macrophages engulf cell debris and dead cells, producing proinflammatory cytokines, chemokines, and reactive oxygen species (ROS) to prevent pathogen colonization and further enhance the recruitment of monocytes/macrophages [55].

Upon recruitment to inflamed tissues, infiltrating macrophages initially adopt proinflammatory phenotypes, driving Th1 cell recruitment and differentiation. In the late stages of inflammation, they transition to an anti‐inflammatory phenotype, producing IL‐10 in the inflammatory environment while promoting wound healing, a process associated with the downregulation of Ly6C expression [56]. This balance—tightly regulated by signaling pathways and various factors—when disrupted, can drive disease pathogenesis. Conversely, CCR2^low^Ly6C^low^CX3CR1^high^ monocytes act as patrolling sentinels, coordinating the clearance of neutrophil debris in the endothelium and phagocytosing particles and cell fragments within capillaries [57]. Upon clearance of debris, neutrophils undergo apoptosis immediately and are cleared by M1‐like macrophages, a process known as efferocytosis [58]. Efferocytosis induces the production of anti‐inflammatory cytokines and GFs such as transforming growth factor‐β (TGF‐β), IL‐10, and vascular endothelial growth factor (VEGF), stimulating the phenotypic transition of proinflammatory macrophages into anti‐inflammatory macrophages (Ly6C^low^CX3CR1^high^) [49, 59]. These macrophages exhibit potent immunosuppressive properties, possess regenerative characteristics, and secrete various angiogenic and GFs, cytokines, and chemokines (e.g., matrix metalloproteinases, platelet‐derived GF resistin‐like molecule α, VEGF, IL‐8, TGF‐β, IL‐10, and arginase), which influence the migration and activation of various cell types and induce the proliferation [60].

Beyond their well‐characterized roles in inflammation, macrophages are essential for maintaining tissue homeostasis through continuous surveillance and renewal. Even in the absence of overt inflammation, circulating monocytes can gradually replenish the macrophage compartment in certain tissues, replacing the embryo‐derived cells (e.g., intestinal macrophages undergo complete replacement every 2–3 weeks in the adult mouse intestine [61, 62]). This physiological turnover, however, becomes dysregulated during aging through two interconnected mechanisms: The first is clonal hematopoiesis of indeterminate potential, which is very common in the elderly and can lead to “inflammation,” causing a 3–5 fold increase in the output of inflammatory monocytes and impaired differentiation, accompanied by accelerated replacement of embryonic‐derived macrophages, which involves the acceleration of human aging and disease. Epidemiological studies have revealed that clonal hematopoiesis is linked to increased risks of all‐cause mortality and age‐related diseases, particularly cardiovascular and CNS pathologies [63, 64, 65, 66, 67]. On the other hand, it is associated with targetable somatic mutations, including expanded blood cell clones with preleukemic driver gene mutations and/or chromosomal abnormalities [63, 66, 67]. The functional and lifespan implications of macrophages developed from clonal hematopoiesis, or those associated with aging, will be revisited later in the discussion. Remarkably, these are pharmacologically targetable, which also hints at potential interventions to restore macrophage homeostasis, which we will mention in a subsequent discussion.

## 3 Classical M1‐Like and M2‐Like Polarization States of Macrophages

**FIGURE 2:**
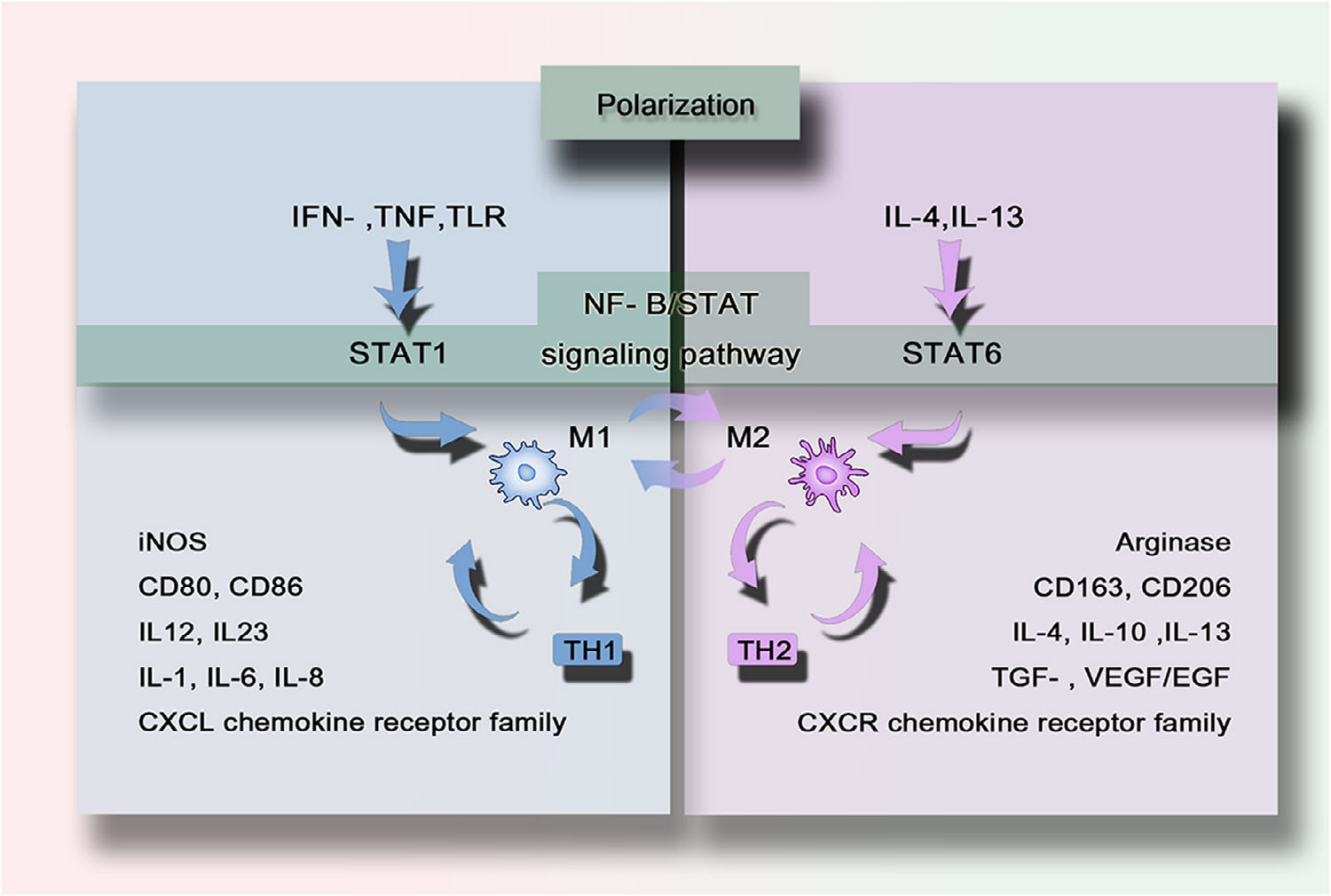
Regulation of macrophage polarization by the NF‐κB/STAT pathway. The NF‐κB/STAT axis integrates microenvironmental signals to determine macrophage functional phenotypes. Red and blue arrows highlight the antagonistic interactions between the M1 and M2 pathways, with NF‐κB serving as a central node for inflammatory signaling. This illustrates how signals derived from pathogens or cytokines dynamically alter macrophage function in the context of disease.

While macrophage developmental origins determine their tissue distribution and longevity (Section 2), their functional specialization is dynamically regulated by polarization states—a continuum bridging embryonic programming and environmental cues. Although single‐cell omics reveals a spectrum of phenotypes, the classical M1‐like/M2‐like dichotomy remains clinically useful for delineating two functional extremes: cytotoxic (M1‐like) and reparative (M2‐like) responses [13, 14]. These polarized states engage in cross‐talk with adaptive immunity, where M1‐like or M2‐like dominance drives T helper (Th) cells, particularly Th1 and Th2 cells, contribute to the formation of a self‐amplifying feedback loop (Figure 2) [68, 69]. By harnessing macrophage plasticity, therapeutic strategies can target the polarization switch (e.g., via nanoparticle delivery) to reprogram macrophages in inflammatory diseases and cancer, enabling precision immunotherapy.

### 3.1 The M1‐Like /M2‐Like Polarization Spectrum: From Antimicrobial Defense to Tissue Repair

M1 macrophage function as a cytotoxic “killing” phenotype, releasing cytokines (e.g., IFN‐γ, TNF) that suppress neighboring cell proliferation and induce tissue damage. Activated by microbial products or Toll‐like receptors (TLRs) ligands, they upregulate antigen presentation, secrete IL‐12/IL‐23, and generate NO and ROS [70]. Polarized M1 macrophages promote Th1 responses and possess potent antimicrobial and antitumor activity. M1‐like phenotype macrophages also produce other proinflammatory cytokines such as TNF‐α, IL‐1β, IL‐6, IL‐8, type I IFN, and CXCL chemokine receptor family members (CXCL1‐3, CXCL5, and CXCL8‐10) [14, 68, 69]. They also express surface markers such as CD80 or CD86, attracting or killing neutrophils and stimulating Th1 responses.

The M2‐like phenotype (alternative activation) promotes tissue repair in sterile healing contexts via cytokine‐mediated proliferation. M2 macrophages do not produce NO or ROS but instead generate ornithine and polyamines through the arginase pathway [71]. M2‐type responses are involved in the suppression of parasitic infections in higher organisms and promote Th2 responses, tissue remodeling, immune tolerance, and tumor progression. Distinctive features of M2 macrophages include upregulation of markers such as dectin‐1, DC‐SIGN, mannose receptor, scavenger receptor A, scavenger receptor B‐1, CD163, CCR2, and CXCR chemokine receptor family members (CXCR1 and CXCR2) [14, 68, 69]. The M2‐like response is associated with the production of TGF‐β, GFs such as VEGF and epidermal growth factor (EGF), and the expression of the cell surface markers CD163 or 206. The M2‐like response can be further amplified by IL‐4, IL‐10, and IL‐13, with a tendency to stimulate Th2 responses such as antibody production [72]. M2‐like skewing facilitates tumor progression or fibrosis but aids wound healing. This plasticity highlights macrophages as therapeutic targets for modulating immune responses.

### 3.2 The Polarization Balance of Macrophages is Associated with Multiple Diseases

These polarization states exhibit tissue‐specific modulation, as exemplified in the liver microenvironment, Chemokines such as CXCR3, CXCL9, CXCL10, and CXCL11 control the infiltration of immune cells during liver injury, thereby affecting hepatic inflammation and fibrosis [73, 74]. Among them, CXCR3 is involved in virus‐related chronic liver inflammation and plays a crucial role in the occurrence of nonalcoholic steatohepatitis by inducing cytokine production, nuclear factor kappa‐light‐chain‐enhancer of activated B cells (NF‐κB) activation, macrophage infiltration, fatty acid synthesis, and T lymphocyte accumulation (Th1 and Th17 immune responses), leading to autophagolysosome damage and endoplasmic reticulum stress [75]. In mice, the use of CXCR3 antagonists to block CXCR3 reverses established steatohepatitis [75]. The chemokine receptor CCR8 is significantly upregulated in injured livers, mediating the recruitment of hepatic macrophages and affecting the trafficking of monocyte/macrophage, monocyte‐derived DCs, and Th cell subsets, thereby influencing the inflammatory response in the damaged liver while also impacting the differentiation of macrophages/DCs and Th cells. CCR8‐deficient animals exhibit increased Th1 polarization of liver CD4(+) T cells and decreased Th2 cell polarization. Inhibiting CCR8 or its ligand CCL1 may represent an important target for protecting the liver from injury, improving initial inflammatory responses, and reducing liver fibrosis [76]. Furthermore, IRF5 has been shown to be significantly induced in hepatic macrophages of both mice and human fibrotic subjects. Transcriptional reprograming in macrophages lacking IRF5 confers immunosuppressive and antiapoptotic characteristics.

In vitro studies indicate that human monocytes during the inflammatory phase can differentiate into the M1‐like phenotype, characterized by inflammatory pathways, followed by maturation into the M2‐like phenotype during the resolution of inflammation, involving pathways associated with metabolism and gene rearrangement [77]. Exposure to classical M1 signals, TLR ligands, or IFN‐γ can also reprogram M2 macrophages to express M1 genes [78]. In summary, the initiation and resolution of inflammation depend on the coordinated proinflammatory and anti‐inflammatory responses of macrophages. During the progression of inflammation, macrophages undergo dynamic transformations and maintain homeostatic balance. When there is a disruption in anti‐inflammatory signals, such as excessive phagocytosis of apoptotic cells by macrophages, the insufficient proinflammatory response can lead to inadequate pathogen clearance, resulting in secondary infections or damage to the affected organs [11]. Furthermore, in mouse models of acute lung injury, inhibition of macrophage depletion has been shown to impair the resolution of acute lung injury [12]. It is important to note, however, that this simple M1/M2 dichotomy does not fully capture the complexity of macrophage polarization in vivo. Nevertheless, this classification remains useful for summarizing and understanding the basic concepts of macrophage function.

The balance between M1‐like and M2‐like polarization of human macrophages is intricately linked to a variety of diseases. Macrophage‐based cellular therapies have demonstrated substantial potential in treating a broad spectrum of illnesses, including autoinflammatory disorders [79, 80, 81] and cancer [82, 83]. For example, IRF5 governs the M1‐like phenotypic activation of hepatic macrophages, promoting hepatocyte death and liver fibrosis in mice and humans. Modulation of IRF5 function may represent an attractive approach for experimental therapy of fibroinflammatory liver diseases [84]. In tumors, M2‐type macrophages foster tumor progression, while M1‐type macrophages impede it [85, 86, 87, 88, 89]. Consequently, employing various strategies, such as targeting immune‐suppressive molecules [90, 91], utilizing bioagent‐laden nanoparticles [92], or deploying monoclonal antibodies [93], to reprogram tumor‐associated macrophages into forms with antitumor activity represents a viable antitumor approach. However, given that the differentiation of macrophages into M1‐like or M2‐like phenotypes is a tightly regulated process involving a network of signaling pathways, transcriptional, and posttranscriptional regulatory mechanisms, achieving a delicate equilibrium is essential. This equilibrium seeks to minimize adverse effects on healthy tissues while averting detrimental immune reactions and effectively curing the disease. In this context, a profound understanding of macrophage‐associated polarization mechanisms and balance conditions is crucial for enhancing the safety and efficacy of macrophage‐based immunotherapies.

## 4 Signaling Pathways Associated with Macrophage Polarization

Macrophage polarization is orchestrated by two core signaling axes: nuclear factor kappa light‐chain enhancer of activated B cells (NF‐κB) and signal transducers and activators of transcription (STATs). IFN and TLR signals induce the classical activation of macrophages, driving them toward the M1‐like phenotype through the NF‐κB/STAT pathway (via STAT1), while IL‐4 and IL‐13 induce the alternatively activated macrophages driving them toward the M2‐like phenotype through the NF‐κB/STAT pathway (via STAT6). Furthermore, macrophage polarization can also be modulated through alternative pathways and stimuli, including metabolic regulators and microenvironmental cues.

### 4.1 TLR and IRF Pathways: Drivers of M1‐Like Polarization

**FIGURE 3:**
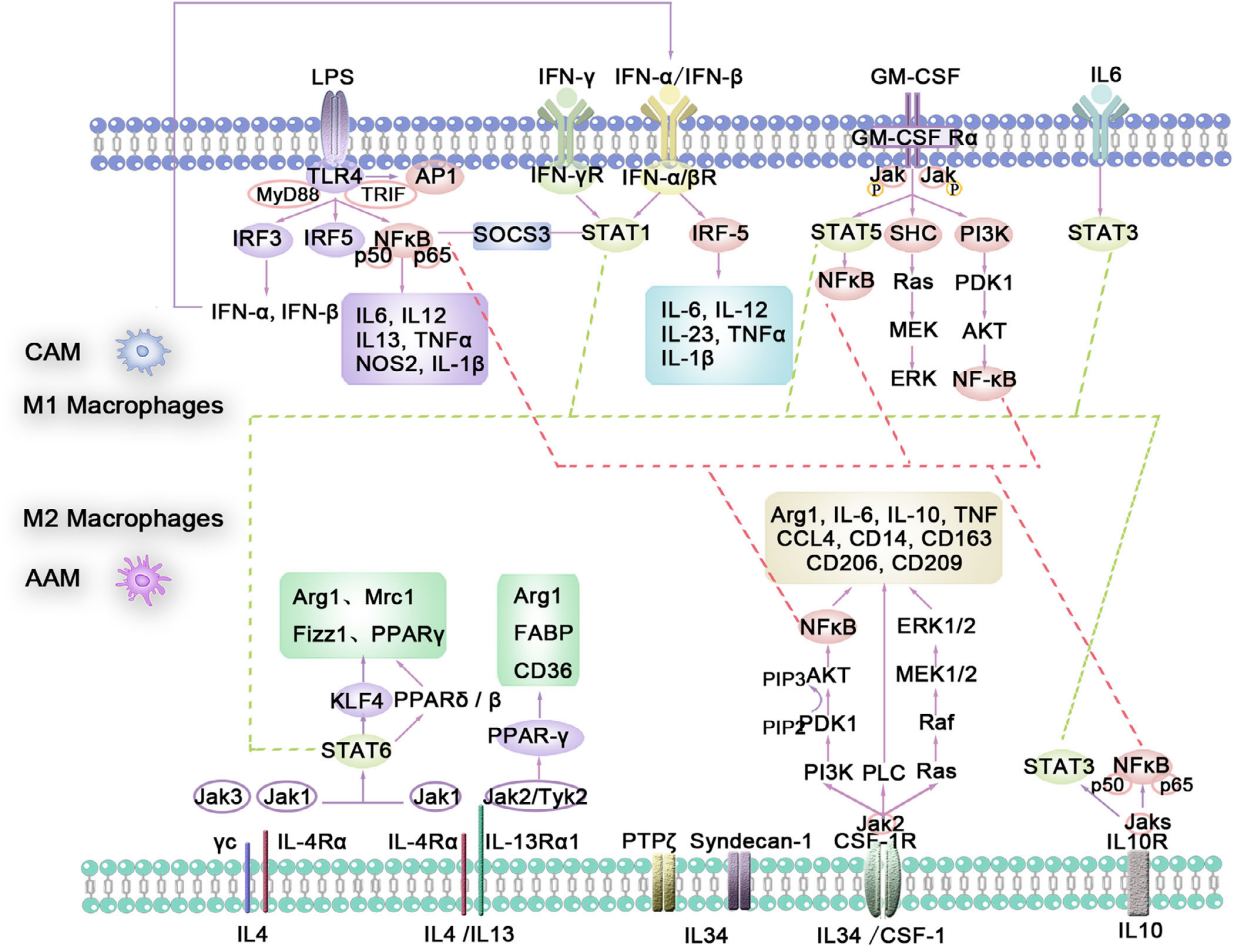
Macrophage M1/M2 polarization mechanisms. Macrophages polarize into M1 or M2 states via distinct pathways: TLR4 ligands activate M1 through MyD88/TRIF and NF‐κB/IRF3, while cytokines like IL‐4/IL‐13 induce M2 polarization via JAK–STAT signaling. IL‐34 and CSF‐1 also promote M2 activation through intracellular cascades.

Exposure to TLR ligands, particularly those stimulated by LPS, other microbial ligands, and IFN‐γ, causes polarization of macrophages (Figure 3). TLR has two adapters, MyD88 and TRIF [94, 95], which mediate the downstream signaling of TLR4. The signaling pathway through MyD88 leads to the activation of a series of kinases, including IRAK4, TRAF6, and inhibitor of kappa B kinase (IKK) [96, 97], resulting in the activation of NF‐κB [95]. This ultimately leads to M1‐like activation and regulation of the expression of a large number of inflammatory genes, increasing the production of proinflammatory cytokines, including TNFα, IL‐6, IL‐12, IL13, NOS2, IL‐1B, and COX2. The survival and polarization of macrophages are closely related to the differential expression of various TLRs. TLR4, TRIF, and MyD88 are crucial for the generation of BM‐derived macrophages and CD11c^+^ adipose tissue macrophages in obese mice [97], whereas TLR4 deficiency promotes the alternative activation (M2) of adipose tissue macrophages [98].

NF‐κB and IRF5 are two major transcription factors that activate the classical activation of macrophages phenotype by regulating the expression of several inflammatory factors, including TNF‐α, IL‐1β, COX2, and IL‐6. The typical NF‐κB pathway is primarily activated by proinflammatory receptors (e.g., TNF receptor superfamily, TLR family) and cytokine receptors (e.g., interleukins). The activated upstream signals converge on the IKK trimeric complex, which consists of two kinases, IKKα and IKKβ, and a regulatory subunit, IKKγ [99, 100]. The activated upstream receptors first phosphorylate and activate IKKβ, which further phosphorylates the inhibitor molecule kappa B(I‐κB), allowing the NF‐κB p65/p50 heterodimer to be released and translocated into the nucleus [101]. Through the TRIF adapter pathway, the transcription factor IRF3 is activated [102], leading to the expression of type I interferons such as IFNα and IFNβ, which bind to the type I interferon receptor to activate the transcription factor STAT1 [14, 72]. IFN‐β may be involved in regulating the expression of IRF1 and IRF5, maintaining the M1‐like polarization state and its functionality [103].

IRFs play a crucial role in hematopoietic development, differentiation into macrophages, and regulation of macrophage maturation, phenotype polarization, phenotype switching, and function [104]. Among the nine IRFs, at least three (IRF‐1, IRF‐5, and IRF‐8) are involved in promoting the proinflammatory M1‐like phenotype, whereas IRF‐3 and IRF‐4 control M2‐like polarization. Among them, IRF5 serves as an important marker for identifying M1 and M2 macrophages [105]. IRF5 collaborates with NF‐κB to provide a cytokine environment that supports Th17 responses, whereas for supporting Th1 responses, IRF5, rather than NF‐κB signaling, is crucial. Lack of IRF5 severely impairs the secretion of IL‐1β, IL‐6, IL‐12, IL‐23, and TNFα, whereas p65 deficiency only impairs the secretion of IL‐6, IL‐12, and IL‐23 [106].

### 4.2 GM‐CSF Stimulates Macrophages to Polarize Toward a Proinflammatory Direction

CSF are a class of cytokines discovered in in vitro studies of hematopoietic cells that are capable of stimulating the proliferation and differentiation of multipotent HSCs and hematopoietic progenitor cells at different developmental stages.

Granulocyte‐macrophage CSF (GM‐CSF, CSF‐2), mainly secreted by T cells, drives BM progenitor cell production and differentiation. GM‐CSF is expressed at low levels in circulation under steady‐state conditions and is induced under inflammatory conditions [107], meaning that it is often considered a proinflammatory cytokine. Compared with M2‐polarized macrophages induced by interleukin‐34 or macrophage CSF (M‐CSF, CSF‐1) mentioned later, GM‐CSF‐induced macrophages exhibit a clear proinflammatory effect, promoting polarization toward the M1‐like direction by expressing proinflammatory cytokines such as TNF and IL‐6 [107], phagocytosis, and a tendency for antigen presentation [108, 109]. Although GM‐CSF and CSF‐1 receptors are unrelated, both interact with Ras‐dependent signaling pathways (Ras–ERK1/2).

Under specific stimulation, the GM‐CSF receptor is specifically activated in the membrane‐proximal region through the JAK2 signaling pathway, leading to tyrosine phosphorylation, without affecting JAK1, JAK3, or TYK2 (the second kinase is tyrosine kinase 2). JAK2 [110], as the major kinase regulating all known activities of GM‐CSF, mediates GM‐CSF‐induced c‐fos activation through receptor phosphorylation and SHC/PTP 1D activation [111, 112]. Phosphorylated JAK2 recruits STAT‐5‐containing SH2 domains, which can also interact with other members of the STAT transcription factor family, including STAT1 and STAT3 subtypes [109, 110, 113], activating the JAK–STAT pathway to control cell differentiation and inflammatory signals. Furthermore, phosphorylated JAK2 on the GM‐CSF receptor can activate phosphoinositide 3‐kinase (PI3K) to initiate the PI3K/AKT pathway. PI3K/AKT signaling requires two metabolites (PIP2/PIP3) and two genes (PTEN/PDK1): pivotal metabolites PIP2 (phosphatidylinositol‐4,5‐bisphosphate) and PIP3 (phosphatidylinositol‐3,4,5‐trisphosphate), and key genes PTEN (phosphatase and tensin homolog) and PDK1 (3‐phosphoinositide‐dependent protein kinase‐1) [114]. Activated PI3K promotes the conversion of PIP2 to PIP3. PIP3 activation of PDK‐1 leads to Akt phosphorylation at the Thr308 site. Activated AKT regulates various cellular biological functions through interaction with numerous downstream signaling molecules such as p21, p27, TGF‐β, ataxin‐1, GABA receptors, Bad, NF‐κB, and mammalian target of rapamycin (mTOR). PTEN can suppress the PI3K/AKT signal and thus inhibit tumorigenesis by dephosphorylating PIP3 to PIP2 [114]. Phosphorylated JAK2 recruits the adapter protein SHC, activating RAS to initiate the MAPK pathway. This cascade induces nuclear signaling and stimulates downstream targets regulating cell growth, proliferation, and differentiation [115].

These three pathways, comprising the JAK2–STAT5, PI3K/AKT, and Ras–MAPK pathways [115, 116], are downstream of the GM‐CSF receptor and interconnected by various factors, although each has a relatively clear role. Research has shown that GM‐CSF is a crucial pulmonary regulatory molecule that plays a key role in maintaining surfactant homeostasis, alveolar stability, lung function, and host defense. GM‐CSF is essential for the maturation of alveolar macrophages, and knockout mice develop pulmonary alveolar proteinosis as a result [117]. Therefore, GM‐CSF represents a promising therapeutic approach for chronic lung diseases, including asthma, chronic obstructive pulmonary disease [118], autoimmune pulmonary alveolar proteinosis [119], idiopathic pulmonary fibrosis, and pulmonary nodules [120, 121]. In addition, GM‐CSF‐induced macrophage polarization plays an important role in the development of many autoimmune and inflammatory diseases, such as rheumatoid arthritis [122, 123], nephritis, and atherosclerosis. Targeting GM‐CSF can be used to treat inflammation and autoimmune diseases. GM‐CSF derived from breast tumor cells can promote the development of an immunosuppressive breast cancer microenvironment by regulating the expression of myeloid cell ARG1 and can also enhance immunotherapy for breast cancer [124].

### 4.3 Macrophages can be Driven Toward an M2‐Like Phenotype Through Typical M2 Stimuli

Macrophages can be driven toward the M2‐like phenotype through typical M2 stimuli such as IL‐4, IL‐13, and IL‐10. IL‐4 or IL‐13 signals initiate through two types of heterodimeric transmembrane receptor complexes: Type I receptors, which bind only IL‐4 and are composed of IL‐4Rα and γ_c_ subunits (the latter also acts as a subunit in IL‐2, IL‐7, IL‐9, IL‐15, and IL‐21 receptor complexes); and Type II receptors, which can bind both IL‐4 and IL‐13 and are composed of IL‐4Rα and IL‐13Rα1 subunits [125, 126]. Therefore, the specificity of the expression of Type I receptor complexes on any particular cell type restricts the responsiveness to IL‐4, whereas the expression of Type II receptors allows signaling for both IL‐4 and IL‐13. Macrophages express both Type I and Type II receptors. Both Type I and Type II receptors initiate signal transduction through JAK/STAT‐mediated phosphorylation events, but differ in specific subunit‐specific adapters: JAK1 binds to IL‐4Rα; JAK2 or Tyk2 is associated with IL‐13Rα1; and JAK3 is associated with γc [127]. Thus, JAK1 and JAK3 are activated through the Type I receptor complex, whereas JAK1 and JAK2 or TYK2 are activated through the Type II receptor complex (Figure 3).

STAT‐polarized macrophages are activated not only by IL‐4 and IL‐13 but also regulated by members of the suppressor of cytokine signaling (SOCS) family. M2 macrophages show selective and IL‐4‐dependent upregulation of SOCS‐1. Enhanced SOCS‐1 promotes the expression of M2‐like characteristics in IL‐4‐induced macrophages, including a higher Arg I/iNOS activity ratio, inhibition of T cell proliferation, diminished response to IFN‐γ/LPS, and reduced SOCS‐3 expression. At the same time, SOCS‐1 also limits the secretion of IL‐10 and Arg I in M1 macrophages.

Upregulation of SOCS‐3 is crucial for effective M1 macrophage activation and function. However, the lack of SOCS‐3 can also promote M1 macrophage polarization and inflammation. SOCS‐3 dually regulates macrophage activation: suppressing LPS‐induced proinflammatory signals while inhibiting IL‐10/STAT3‐mediated anti‐inflammatory feedback [128, 129]. It does so by controlling NF‐κB activation and nuclear accumulation, as well as PI3K activity, to inhibit the expression of anti‐inflammatory IL‐10 and SOCS‐1, suppressing IL‐10‐triggered STAT3 tyrosine phosphorylation [128], and driving the production of macrophage proinflammatory factors such as IL‐1β, IL‐6, IL‐12, IL‐23, and NO [130].

IL‐4 /IL‐13 activate PPARδ/β via STAT6‐binding promoter elements, driving M2‐like polarization [131]. The nuclear receptors PPARγ [132] and PPARδ [131, 133] control different gene subgroups related to the activation and oxidative metabolism of M2 macrophages. The alternative activation (M2a) of resident macrophages in the liver and adipose tissue highly depends on the activity of PPARδ [133]/PPARγ [132]. PPARγ agonists such as abscisic acid can induce adipose tissue‐resident macrophages to polarize toward an alternative M2‐like phenotype. This induction leads to the expression of Arg1 and various transport proteins (FABP, CD36), resulting in improvements in fatty acid metabolism, insulin sensitivity, and obesity‐related inflammation [132, 133, 134].

Krüppel‐like factors (KLF2and KLF4), zinc finger transcription factors, regulate M2‐like polarization. KLF4 is induced by IL‐4 via STAT6, directly promoting alternative activation (M2) in vitro and in vivo [135]. KLF4 is strongly induced in M2 macrophages and significantly reduced in M1 macrophages. In coordination with STAT6 and PPARγ, KLF4 promotes the expression of M2‐like genes (Arg‐1, Mrc1, Fizz1, PPARγ) and inhibits M1 genes (TNFα, Cox‐2, CCL5, iNOS). KLF2 has emerged as a transcription factor involved in various inflammatory diseases, where it functions to regulate immune cell function and inflammation mediated by NF‐κB [136, 137]. Macrophages with KLF4 deficiency show increased expression of proinflammatory genes, enhanced bactericidal activity, and altered metabolism [135].

The overexpression of KLF2 suppresses IL‐1β‐induced apoptosis and matrix degradation by inhibiting ROS production [138] and acts as an effective inhibitor of NF‐κB‐dependent hypoxia‐inducible factor transcription [139]. Emerging evidence suggests KLF4 [140] or KLF2 [141] activation mitigates tissue damage and inflammation in osteoarthritis [138].

### 4.4 CSF‐1 Activates CSF‐1R, which in Turn Activates AKT via the PI3K Pathway

CSF‐1 critically regulates macrophage homeostasis under physiological conditions. In mice and humans, CSF‐1 alone can induce the differentiation of macrophages from an antigen‐presenting phenotype to an immune suppressive phenotype [142], similar to M2‐like activated macrophages [143], as they can still respond to M2‐inducing lymphokines, such as IL‐4, and express related genes, such as IL‐6, IL‐10, TNF, CCL4, CD14, CD163, and CD209 [143, 144]. CSF‐1 is also considered an effective inducer of M2. CSF‐1R comprises a unique α‐chain and a β c subunit shared with IL‐3 and IL‐5 receptors [145]. The distal C‐terminus of the β subunit couples with the Ras–ERK1/2 [146, 147]. After binding to the extracellular domain of CSF‐1R, CSF‐1 or IL‐34 induces dimerization and tyrosine kinase‐mediated cytoplasmic tyrosine residue phosphorylation. Phosphorylated tyrosine residues interact with JAK2 and activate JAK2 [148], thereby activating various intracellular signaling pathways through the docking of specific SH2 domain proteins, including Ras, PI3K, and phospholipase C, resulting in cascading intracellular signaling responses [149, 150, 151, 152, 153, 154].

In macrophages, activated CSF‐1R activates AKT through the PI3K pathway directly or indirectly, such as via the ceramide‐1‐phosphate pathway [155]. The Ras–ERK1/2 couples with the distal C‐terminus of the β subunit [146, 147], where the activated Ras pathway regulates macrophage differentiation by controlling the activity of downstream nuclear targets such as transcription factors activating protein‐1. p53 and STAT3 may generate crosstalk through coordinated regulation by the MAPK and PI3K/AKT signaling pathways, which is known to affect cancer progression and metastasis [156]. In myeloid progenitor cells, the downstream activation of CSF‐1R initiates STAT3 and activates ERK1/2, leading to PP2A inactivation, which plays a central role in CSF‐1‐induced differentiation. Modulation of the ERK1/2 pathway can inhibit tumor‐associated macrophage M2‐like polarization [157].

The PI3K/AKT and Ras/ERK1/2 pathways play crucial roles in CSF‐1‐mediated macrophage survival. The CSF‐1R pTyr807 signal promotes macrophage proliferation and differentiation independently by activating the Ras and PI3K pathways, whereas the ceramide‐1‐phosphate pathway produced under CSF‐1 induction indirectly stimulates proliferation by activating the PI3K/AKT, JNK, and ERK1/2 pathways [155].

The CSF‐1–CSF‐1R signaling pathway may deplete tumor‐associated macrophage and myeloid‐derived suppressor cells responsible for the immunosuppressive tumor microenvironment (TME) [158]. The interaction between CSF‐1 and glial progenitor cells enhances the invasion of glioblastoma (GBM), while inhibition of CSF‐1R targeting glioma‐associated microglia may suppress GBM invasion [159]. Depletion of tumor‐associated macrophages in neuroblastomas may be associated with increased chemotherapy efficacy [160]. CSF‐1R inhibition significantly reduces F4/80^+^ tumor‐associated macrophage while increasing the ratio of CD8^+^/CD4^+^ T cells [161]. In addition, inhibiting CSF‐1R can prevent monocytes recruited in GBM from differentiating into immunosuppressive, proangiogenic M2 macrophages [162]. The selective CSF‐1R inhibitor vimseltinib blocks CSF‐1R signaling, reducing M2‐like tumor‐associated macrophages. This action effectively reduces the number of M2‐type tumor‐associated macrophages, thereby alleviating immunosuppression within the TME and promoting antitumor immune responses. The drug is currently approved for the treatment of patients with advanced solid tumors and tenosynovial giant cell tumor, especially those experiencing significant symptoms or joint dysfunction due to tumor growth and who are not suitable candidates for surgical resection. Its mechanism of action involves reducing CSF‐1‐driven macrophage infiltration in the TME, thereby significantly alleviating tissue proliferation and inflammatory responses [163, 164, 165]. In addition, therapeutic drugs targeting CSF‐1 secretion in melanoma have been shown to reduce the expansion of the monocyte–myeloid‐derived suppressor cells subset and macrophage conversion, reprogram regulatory myeloid cells, and reduce tumor progression [166].

### 4.5 IL‐34 can Completely Substitute for CSF‐1 in Inducing Monocyte to M2‐Like Polarization via AMPK

While most BM‐derived populations primarily rely on CSF‐1 signaling through CSF‐1R for their development and survival, some self‐renewing tissue macrophages, particularly microglia, require tissue‐restricted signals from the alternative ligand IL‐34 [167, 168]. When exposed to various inflammatory stimuli (e.g., proinflammatory cytokines, pathogen‐associated molecular patterns, and chemical stressors), NF‐κB is activated, leading to the induction of IL‐34 expression in multiple cell types [168]. As a tissue‐specific ligand for CSF‐1R, IL‐34 induces activation of the ERK1/2 and AKT signaling pathways and has the potential to amplify the inflammatory cycle by inducing the expression of various proinflammatory cytokines, chemokines, and metalloproteinases in multiple cells, including monocytes and macrophages, acting in tissue‐resident macrophages [169]. By contrast, IL‐34 can also act as an immunosuppressive cytokine, leading to strong activation of the AMPK signaling pathway, which can fully replace CSF‐1 induction of monocytes toward M2‐like polarization, exhibiting anti‐inflammatory properties [170, 171]. IL‐34 dose dependently induces M2‐like markers (CD206, IL‐10 [167]) in monocyte‐derived macrophages (MoMFs) while downregulating TLR2/dectin‐1 [172], thereby suppressing NK/T‐cell responses to promote immune tolerance.

IL‐34 additionally binds protein tyrosine phosphatase zeta (PTPζ) and CD138 (Syndecan‐1)—receptors expressed in epithelial cells, the CNS, and cancers [173], This interaction drives Syndecan‐1‐dependent migration of THP‐1 monocytes and M2a macrophages. IL‐34 also shifts and polarizes Kupffer cells toward an M2‐like phenotype, reducing the expression of proinflammatory cytokines such as IL‐12, while increasing the expression of immunosuppressive cytokines such as IL‐10, and TGF‐β1, inducing activation of the PI3K/AKT pathway, enhancing mTOR phosphorylation, and suppressing p65 and p38 MAPK activation [174]. In osteoclasts, similar to CSF‐1, IL‐34 coregulates the differentiation and survival of osteoclasts with the receptor activator of NF‐κB, activating downstream signaling pathways of CSF‐1R to regulate osteoclast precursor cell adhesion, differentiation, fusion, and resorptive activity [175, 176]. Overall, IL‐34 may play a crucial role in coordinating innate and adaptive immune responses by modulating the expression of cytokines and chemokines and by polarizing macrophages into different phenotypes.

Dysregulation of the CSF‐1/IL‐34–CSF‐1R axis contributes to immune pathologies through two mechanisms. Increased M2 macrophages enhance the proliferation of malignant pleural mesothelioma cells and increase the resistance to treatment in pleural effusions of patients [177, 178]. The CSF‐1/IL‐34–CSF‐1R pathway is also related to chronic inflammation, oxidative stress, ROS generation, and sustained abnormal signal transduction [179]; thus, regulation of CSF‐1R may hold promise for the treatment of chronic inflammatory diseases [180].

IL‐34 and CSF‐1 share structural homology and compete for CSF‐1R binding, yet diverge in downstream signaling and organ‐specific expression patterns [181, 182]. In renal tubular cells, both activators of CSF‐1R are released during acute injury, with CSF‐1 promoting tubular cell survival and kidney repair, and IL‐34 promoting chronic kidney injury [183]. IL‐34‐dependent macrophage‐mediated mechanisms promote acute kidney injury induced by sustained ischemia, which can worsen subsequent chronic kidney disease [184], suggesting that altering the balance of these factors may be an effective approach to improve the prognosis of acute kidney injury. The expression patterns of IL‐34 and CSF‐1 in the small intestine and colon differ, with higher levels of IL‐34 in the small intestine and higher levels of CSF‐1 in the colon [185]. In humans, infiltrating cells expressing IL‐34 in the innate layer and intestinal epithelial cells, along with TNF‐α, regulate the expression of IL‐34 in intestinal epithelial cells through the NF‐κB pathway [186]. In inflamed intestinal tissues, the expression of IL‐34 and CSF‐1 increases with inflammation, and the specific dysregulation of macrophage genes is associated with genetic susceptibility to chronic inflammatory bowel disease [185, 186], suggesting that IL‐34 and CSF‐1 are novel regulators of inflammatory bowel disease inflammation.

### 4.6 Other Factors, such as IL‐10 and IL‐6, also Participate in Macrophage Polarization

IL‐10 and IL‐6 exhibit distinct biological activities despite both recruiting JAKs3 and activating STAT transcription factor receptors, primarily STAT3 [187, 188]. IL‐6 possesses both pro‐ and anti‐inflammatory activities [187], such as stimulating T‐cell proliferation, differentiation into cytotoxic T‐cells, and inducing antibody production, while IL‐10 is a potent anti‐inflammatory cytokine that plays a crucial role in inflammation and immune responses. STAT3 recruitment mainly occurs in macrophages. Upon binding of IL‐10 to IL‐10R, phosphorylated JAKs activate the IL‐10RI subunit on IL‐10R, creating docking sites for STAT transcription factors such as STAT3. Through tyrosine phosphorylation, STAT3 is activated at the receptor and subsequently translocates to the nucleus to induce STAT3‐responsive genes [189, 190]. IL‐6 binds to the IL‐6Rα subunit and the signal transducer gp130 [191]. Similar to IL‐10 signaling, gp130 activation leads to JAK activation, receptor phosphorylation, and ultimately the activation of the transcription factor STAT3 [191].

IL‐10 promotes M2‐like polarization by inducing the activity of the p50 NF‐κB homodimer and activating the JAK–STAT signaling pathway [190], effectively inhibiting the release of proinflammatory cytokines, such as TNF‐α, from macrophages [192]. IL‐10 also induces the synthesis of IL‐1β receptor antagonist and soluble TNF receptors, terminating the inflammatory response. IL‐10 plays a significant role in autoimmune diseases such as multiple sclerosis and systemic lupus erythematosus [193]. Furthermore, both IL‐6 and IL‐10 can induce the production of SOCS‐3 [187, 194], which inhibits IL‐6 signaling through the SH2 domain of gp130 [195]. IL‐10 can induce expression of SOCS‐3 mRNA in human monocytes and neutrophils, suggesting that its ability to inhibit LPS‐induced proinflammatory gene expression may depend on the induction of SOCS‐3 [128]. The responsiveness of neutrophils to IL‐10 is largely dependent on the expression level of IL‐10R1, which is assessed through STAT3 tyrosine phosphorylation, SOCS‐3 expression, and cytokine production [196].

The polarization is also influenced by various inflammatory modulators, signaling molecules, and transcription factors. Specialized or polarized T cells (Th1, Th2, Tregs) play crucial roles in macrophage polarization activation, as mentioned earlier, with M1‐like and M2‐like exerting a positive feedback promotion with Th1 and Th2, respectively. Macrophage polarization is also regulated by local microenvironmental conditions such as hypoxia. Immune complexes can profoundly affect the functional state of macrophages.

## 5 Macrophages and Immunomodulatory Functions

Although macrophages undergo a well‐defined transition from a proinflammatory (M1‐like) to a reparative (M2‐like) phenotype during acute inflammation (as described in Section 3), this balance is often disrupted in chronic pathological conditions. In such settings—particularly those involving persistent low‐grade inflammation—macrophages in various organs exhibit varying degrees of dysfunction or altered behavior. Prolonged stress factors, such as DNA damage, oxidative stress, and the accumulation of the senescence‐associated secretory phenotype (SASP), hijack classical signaling pathways (e.g., NF‐κB, p38 MAPK), reprogramming macrophage behavior and driving a maladaptive shift from immune surveillance toward disease‐promoting phenotypes. In these pathological states, macrophage dysregulation—manifesting in their origin, polarization, and function—is driven by both tissue‐specific microenvironmental cues and systemic signals. Understanding this dysregulation opens new avenues for targeting macrophages in the treatment of chronic inflammatory and degenerative diseases, with potential applications in cancer and autoimmune disorders as well.

### 5.1 Various Factors Exert Influence on the Functions of Macrophages

One prominent example is the phenomenon known as inflammaging, a sterile, low‐grade chronic inflammatory state that progressively intensifies with age. This systemic process affects every cell, tissue, and organ in the body, leading to cellular dysfunction, immune dysregulation, and ultimately, maladaptive cellular changes. While inflammaging reflects a broader immunological shift associated with aging, its impact is particularly pronounced in macrophages, which play central roles in both innate immunity and tissue homeostasis. Various stressors—such as ultraviolet radiation [17, 197], disease, psychological or physiological stress, smoking, or oncogene activation [198]—can trigger DNA damage responses that activate multiple downstream signaling pathways critical for macrophage function. These include NF‐κB [199], p38 MAPK [200], PKD1, and GATA4 [201]. Notably, these pathways not only accelerate cellular senescence but also significantly alter macrophage polarization and function, driving them toward phenotypes that either exacerbate or attempt to resolve chronic inflammation.

Among these, the NF‐κB pathway plays a central role in regulating the SASP—a complex profile of secreted factors produced by aging or damaged cells [199, 201, 202]. NEMO, the regulatory subunit of the IκB kinase complex, is essential for NF‐κB activation. Upon DNA damage, NEMO shuttles between the cytoplasm and nucleus, phosphorylating IκB and initiating NF‐κB signaling, which in turn upregulates the expression of multiple inflammatory cytokines [203, 204]. Additionally, other pathways such as p38 MAPK and RIG‐I are involved in oxidative stress and mitochondrial damage, further activating senescence‐related pathways like p16, p21, and p53 [201, 205], and ultimately promoting SASP production and secretion [204, 206]. The SASP includes a wide array of molecules—such as cytokines (e.g., IL‐1α/β, IL‐6, IL‐8, MCP‐2, MIP‐1α), chemokines (e.g., CCL2, CXCL14), GFs (e.g., EGF, VEGF), and matrix metalloproteinases (e.g., MMP1, MMP3)—that can profoundly influence macrophage behavior [7, 207]. For instance, these factors may reduce macrophage phagocytic capacity and impair their chemotactic response. Interestingly, SASP overstimulation may trigger negative feedback (e.g., via microRNA) to downregulate IL‐6/IL‐8 secretion [208].

**FIGURE 4:**
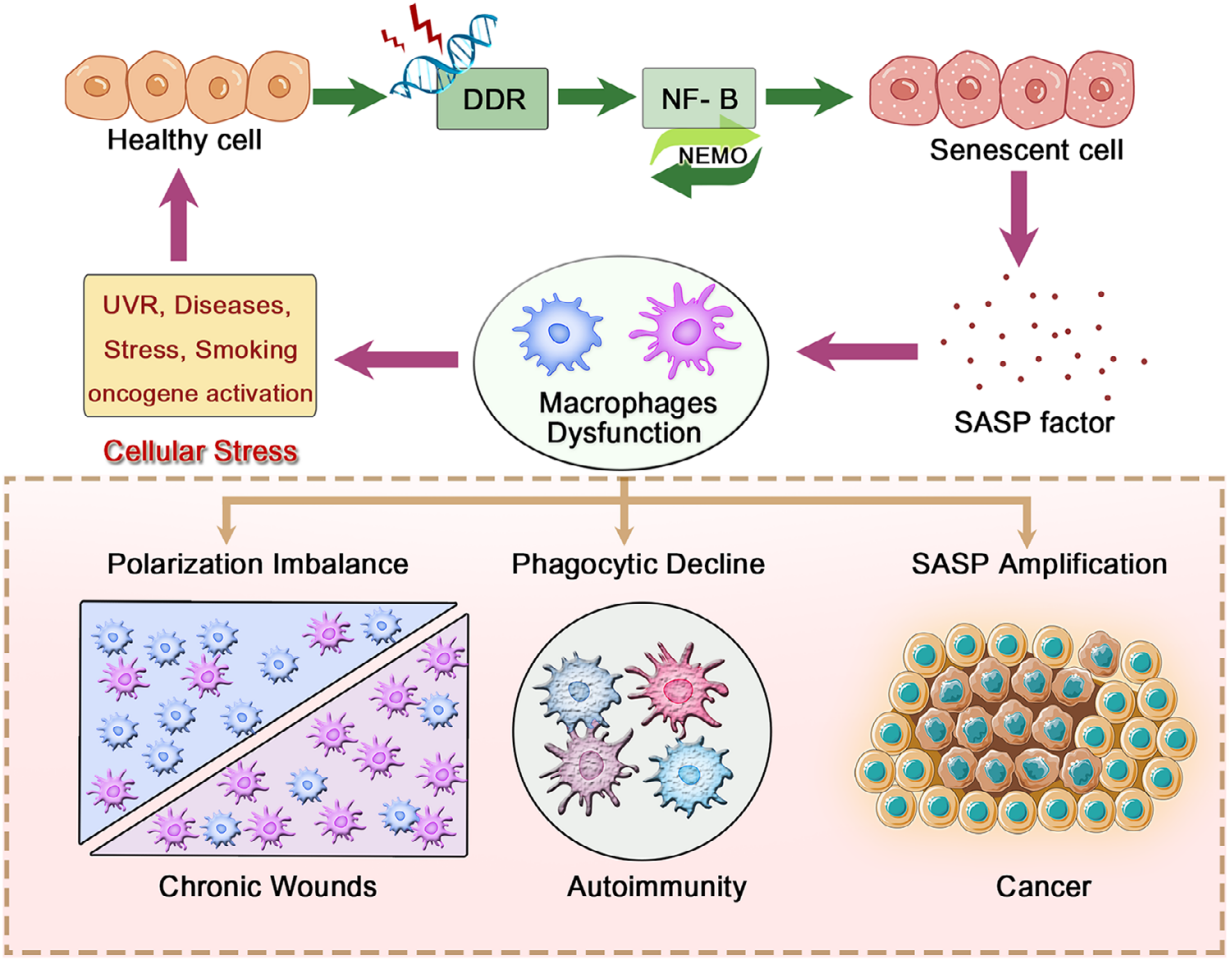
Macrophage dysfunction in inflammation and disease. Stress triggers DNA damage response via ATM, activating NF‐κB. Senescent cells release SASP, impairing macrophage function—causing polarization imbalance, reduced phagocytosis, and chronic inflammation—linked to wounds, autoimmunity, and cancer.

Age‐associated TLR alterations modulate endothelial and immune cell phenotypes via negative feedback loops that curb excessive inflammation [209, 210]. When senescent cells or SASP factors exceeds macrophage clearance capacity, polarization‐induced dysfunction impairs immune surveillance and accelerates senescent cells accumulation [211]. Aging also inhibits the de novo synthesis of NAD^+^ in macrophages, causing them to shift toward a proinflammatory phenotype [212] (Figure 4).

Studies in mice have demonstrated that monocytes and macrophages isolated from aged animals show significantly reduced secretion of IL‐6 and TNF‐α in response to identical in vitro stimuli [213, 214]. This defect has been linked to abnormal TLR mRNA expression [213]. Furthermore, when exposed to conditioned media from aged thyroid cells, macrophages adopt an anti‐inflammatory M2‐like phenotype—characterized by high CD206 and low major histocompatibility complex (MHC) II expression, along with increased CCL17 secretion—which paradoxically promotes tumor progression [215]. Despite the lack of significant differences in total circulating monocyte counts between young and old individuals, notable shifts occur in monocyte subset composition and function. For example, all four major monocyte subsets in older adults produce significantly less IL‐6 and TNF‐α, aligning with the overall reduction in proinflammatory activity seen in inflammaging [214, 216]. Given that classical monocytes possess distinct homing and differentiation potentials based on their hematopoietic origin, alterations in the tissue microenvironment may dictate the recruitment and functional fate of specific MoMFs [47, 217]. Importantly, this age‐associated macrophage dysfunction is not necessarily irreversible. Evidence shows that when removed from the aged microenvironment—either in vitro or through targeted interventions in vivo—these macrophages can regain functionality resembling that of younger counterparts. This highlights the remarkable plasticity and adaptability retained by macrophages even in advanced age [217, 218].

These inflammation‐driven changes in macrophage behavior mirror those observed in other chronic inflammatory diseases such as cancer and diabetes [219, 220, 221]. These pathologies share macrophage dysregulation—in origin, polarization, and function—driven by tissue microenvironment and systemic cues. This understanding opens new avenues for macrophage‐targeted therapies [217]. For instance, combining IL‐2 with anti‐CD40 antibodies has shown promise in enhancing both innate and T‐cell‐mediated immune responses in aged hosts, potentially reversing immunosenescence [217]. Beyond age‐related conditions, these macrophage‐targeted intervention strategies demonstrate broad therapeutic potential across a range of disease contexts. They hold promise not only for treating chronic inflammatory and degenerative disorders, but also for addressing diseases such as cancer and autoimmune conditions. The following subsections will further elaborate on tissue‐specific macrophage populations and outline how tailored therapeutic approaches can be designed to target macrophages in the context of specific diseases.

### 5.2 Skin Macrophages Exhibit a Proinflammatory Tendency During Inflammaging

#### 5.2.1 Functions of Macrophages in the Skin Under Homeostasis

As the body's largest defense organ, the skin serves as both a barrier against harmful stimuli and an immune sentinel, providing a paradigmatic model for studying inflammation‐related macrophage dysfunction [222]. Skin macrophages, as key immune cells in the skin, play a crucial role in maintaining skin homeostasis by inducing apoptosis in aging fibroblasts and clearing dead cells [223]. However, this homeostatic capacity declines with aging, disrupting the precise regulation of inflammatory response. To understand how this regulation is disrupted, we first need to observe the normal behavioral patterns of macrophages in acute inflammation.

By integrating single‐cell RNA sequencing (scRNA‐seq) and spatial transcriptomics (ST‐seq) analyses, we found that during acute inflammation, such as in the process of human skin wound healing after injury, macrophages exhibit stage‐specific activation patterns, playing a particularly critical role during the inflammatory phase [224]. In the early stages of wound healing, proinflammatory macrophages transiently increase in proportion, marked by the upregulation of HIF1α and proinflammatory cytokines (e.g., TNF‐α, IL‐1β, and CCL2). Meanwhile, markers of proresolving macrophages (MRC1, IL‐10, TGF‐β, and PDGFB) are downregulated in the early phase [225, 226]. This temporal shift is key to effective tissue repair.

Traditionally, this transition was thought to rely on the linear differentiation of monocytes. However, recent studies reveal more complex plasticity: As noted in Part 1, it was traditionally believed that Ly6C^hi^ classical (inflammatory) monocytes differentiate into proinflammatory Ly6C^hi^ macrophages, representing a singular differentiation pathway [50, 53, 54, 56]. However, new evidence shows that under specific conditions, Ly6C^hi^PD‐L2^low^ classical monocytes recruited to allergic skin lesions sequentially differentiate into Ly6C^lo^PD‐L2^hi^ proresolving macrophages via intermediate Ly6C^hi^PD‐L2^hi^ macrophages (rather than through Ly6C^low^ nonclassical monocytes), in an IL‐4 receptor‐dependent manner [227]. During this differentiation, macrophages derived from classical monocytes display anti‐inflammatory signatures alongside metabolic rewiring, enabling them to phagocytose apoptotic neutrophils and allergens, thereby contributing to the resolution of inflammation. Such plasticity is critical for balanced inflammation resolution.

Furthermore, the spatial localization of this plasticity further underscores its functional importance. Spatial analysis reveals that during the peak of inflammation, proinflammatory macrophages are located in the upper dermis adjacent to migrating epithelial cells, forming an immune cell cluster with neutrophils, type 3 DCs, and Th cells. These proinflammatory macrophages promote keratinocyte migration and re‐epithelialization by secreting chemokines (e.g., CXCL1 and CXCL5) and EGF receptor ligands (e.g., EREG). Additionally, they participate in constructing a complex intercellular communication network, including autocrine signals (e.g., TGFA, AREG, and HB‐EGF) and paracrine signals (e.g., EREG). These signals work synergistically to enhance FOS‐like antigen 1 expression and promote keratinocyte migration, thereby supporting the inflammatory phase of wound healing [225].

In summary, proinflammatory macrophages significantly promote cell migration and re‐epithelialization during tissue repair through specific polarization states and secreted factors during the inflammatory phase of wound healing, ensuring the balance between inflammation and repair through precise spatiotemporal regulation. Notably, this spatiotemporal precision deteriorates during aging, as chronic inflammatory microenvironments (e.g., SASP‐rich niches) skew macrophages toward pathogenic states—altering their quantity, maturity, and functional polarization (Section 5.1).

#### 5.2.2 Pathological Consequences of Polarization Imbalance

Exposure to both internal (physiological processes) and external factors (e.g., ultraviolet radiation, alcohol consumption, malnutrition, pollutants, or smoking) can lead to skin aging [228]. Aging skin cells exhibit nuclear DNA damage, ROS generation, and release of SASP components, accompanied by structural and physiological impairments [229, 230, 231, 232, 233]. In the epidermis, the transcriptional activity of NF‐κB increases with age, leading to the upregulation of inflammatory cytokines [203, 204, 234]. Increased activation of the NF‐κB pathway results in severe widespread dermatitis and elevated levels of TNF‐α mRNA [235]. Indeed, human skin fibroblasts obtained from elderly individuals have been shown to produce higher levels of proinflammatory cytokines (IL‐1β, IL‐6, IL‐8, and TNFα [221]) upon CMV infection or LPS exposure compared with those obtained from younger individuals, with IL‐6 showing particularly significant differences [236]. This increase in proinflammatory cytokines further leads to the accumulation of proinflammatory M1 macrophages and a decrease in anti‐inflammatory M2 macrophages, which are key cells in normal wound healing [59, 167, 222, 237], and efferocytosis [43, 59]. In this case, the total number of macrophages remains unchanged, but the quantity of IL‐34+ cells is decreased in sun‐exposed aged skin. This suggests age‐related shifts in macrophage subsets arise from altered differentiation, not recruitment [167]. Moreover, the number and maturity of epidermal Langerhans cells is significantly decreased in elderly mice, the dermis of aged skin contains more neutrophils and mast cells compared with that of young individuals, which is associated with the upregulation of IL‐8 [230].

**FIGURE 5:**
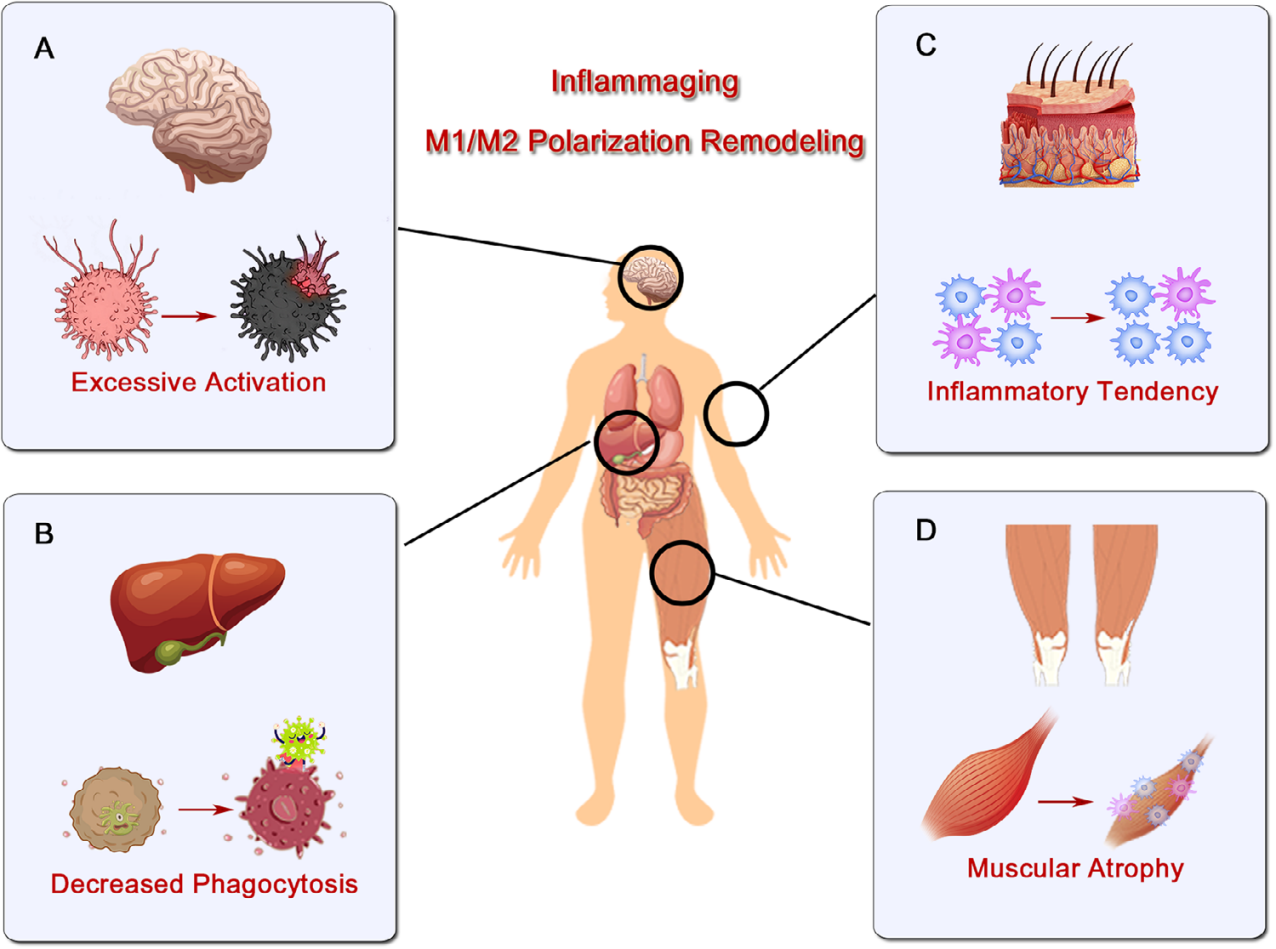
The pathological changes of macrophages in different organs under the state of “inflamaging.” (A) In the brain, remodeling of M1/M2 polarization leads to abnormal neuronal excitation. (B) In the liver, reduced phagocyte function weakens the organ's immune defense capabilities, increasing susceptibility to infections. (C) In skin tissue, an inflammatory tendency manifests as vasodilation and leukocyte infiltration, potentially contributing to the development of chronic dermatological conditions. (D) In skeletal muscle, inflammatory cytokines acting on muscle fibers induce atrophy characterized by reduced muscle volume and diminished strength.

Impairment of macrophage phagocytic function may cause neutrophils to accumulate within the wound, leading to delayed wound healing and scar formation. For instance, the hyperglycemic microenvironment in diabetic patients can lead to delayed macrophage infiltration and decreased phagocytic ability [51, 52], which directly or indirectly causes disturbances in efferocytosis and further results in impaired transition from a pro‐ to anti‐inflammatory phenotype [54, 238]. Cumulatively, these factors ultimately contribute to sustained inflammation and tissue damage [60]. In both acute and chronic loss of epidermal barrier function, there is stimulation of the production of inflammatory cytokines and chemokines in the serum [239], infiltration of inflammatory cells [240], and maturation and proliferation of Langerhans cells [241]. This may lead to interrupted wound healing, placing the body in a state of inflammation, increases susceptibility to chronic inflammatory diseases such as cancer, type 2 diabetes, cardiovascular diseases, osteoporosis, neurodegenerative diseases such as AD, and frailty syndrome [219, 220, 221] (Figure 5).

We also observed inflammaging affects macrophage polarization and function through various pathways at the single‐cell resolution level [18], and in turn, macrophages influence skin cell aging through changes in polarization and function. The supernatant from M1 macrophages increased the percentages of senescence‐associated β‐galactosidase‐positive cells, suggesting a prosenescence effect. In contrast, the supernatant of M2 macrophages decreased the percentage of senescence‐associated β‐galactosidase‐positive cells in vitro, possibly indicating an inhibitory capacity against dermal fibroblast aging [167]. Furthermore, macrophages control the quantity and quality of collagen matrix by regulating the balance between M1 and M2 macrophages, thereby influencing chronic inflammation. Increasing the M1/M2 ratio in the dermis, rather than recruiting M1 macrophages, can induce skin inflammation implicated in skin photoaging [167, 242]. Aging Langerhans cells impair the proliferation of OVA‐specific CD4^+^ and CD8^+^ T cells [243]. The phagocytic function of aged macrophages decreases, leading to the accumulation of undigested debris, exacerbating chronic sterile inflammation and tissue aging. This age‐related imbalance in the proportions of macrophage polarization subpopulations eventually leads to weakened immune responses to pathogens and an increased incidence of chronic inflammatory conditions and autoimmune diseases, such as psoriasis [244, 245], vitiligo [246], rosacea, cutaneous lupus erythematosus, melanoma [247], and nonmelanoma skin cancers [248].

#### 5.2.3 Therapeutic Intervention Strategies for Macrophages in the Skin

Macrophage polarization modulation represents a novel therapeutic strategy. For instance, age‐associated Langerhans cell dysfunction involves miRNA‐mediated regulation of both TGF‐β‐dependent and independent pathways [243]. Topical barrier repair (e.g., moisturizers) in aging skin, can significantly enhance epidermal barrier function and hydration of the stratum corneum, thereby reducing circulating proinflammatory cytokine levels in the elderly and potentially mitigating the downstream progression of chronic inflammatory diseases [221]. In the single‐cell transcriptomic analysis of bullous pemphigoid, immune‐stromal crosstalk drives type 2 inflammation, with macrophages promoting Th2 immune responses via IL‐4/IL‐13 secretion, exacerbating disease progression [249]. Targeting M2 macrophage proinflammatory phenotypes could be a potential strategy to alleviate such conditions. Similarly, in pathological scar formation studies, TEM1/endosialin/CD248 enhances TGF‐β signaling to promote fibroblast activation, while macrophages amplify this fibrotic effect through paracrine actions [250]. Inhibiting the profibrotic phenotype of M2 macrophages might reduce scarring; regulating key genes like STAT6 and PPARγ via drugs or gene editing could modulate macrophage polarization and minimize scar formation. Moreover, Yes‐associated protein (YAP) and IL‐33 in macrophage regulation are closely linked, particularly in inflammation, immune modulation, and the TME. Notably, YAP also plays a critical role in age‐related macrophage dysfunction, where dysregulated mechanotransduction and chronic inflammation contribute to impaired tissue repair and senescence‐associated pathologies [251]. Manipulating macrophage mechanosensing capabilities can alter their functional state to favor tissue regeneration over fibrosis. In large animal models, inhibiting YAP/TAZ or integrin signaling reduces activation of fibrotic M2 macrophages and boosts regenerative M1 macrophages, facilitating scarless healing [224]. Intervening in macrophage mechanical microenvironments using drugs or biomaterials, or employing CRISPR‐Cas9 gene editing of polarization‐related genes (e.g., STAT3, STAT6), provides innovative approaches for developing macrophage‐based therapies.

Therapeutic targeting of these signaling pathways—particularly the JAK–STAT and AHR axis has shown clinical efficacy [252, 253]. JAK inhibitors such as upadacitinib (selective JAK1 inhibitor), abrocitinib (preferred JAK1 inhibitor), and baricitinib (A JAK1/2 inhibitor) have been proven to rapidly alleviate itching and skin lesions in atopic dermatitis, and surpassing conventional immunosuppressants [254, 255]. Meanwhile, the AHR axis enhances skin barrier function by promoting Nrf2‐mediated antioxidant responses and repairing mitochondrial dysfunction, thereby reducing atopic dermatitis recurrence [253]. Clinical trials of AHR agonists like tapinarof have demonstrated significant improvements, with additional benefits in modulating the skin microbiome, indirectly enhancing barrier function. Combining AHR agonists with JAK inhibitors may also synergistically suppress Th2 inflammation and promote barrier repair. In this context, benvitimod emerges as a novel therapeutic agent. By activating the AHR/ARNT pathway, it upregulates tight junction protein expression while inhibiting STAT6 phosphorylation, achieving dual anti‐inflammatory and barrier repair effects [252]. Compared with corticosteroids or single‐target JAK inhibitors, benvitimod offers a more comprehensive strategy for managing atopic dermatitis and is particularly suitable for early intermittent use to prevent recurrence (Table 1).

**TABLE 1:** Macrophage‐targeting therapeutic strategies in clinical trials.

Pathway mechanism	Related drugs	Effects on macrophages	Targeted diseases	Trial ID	Phase	Last update date	Status
CSF‐1R Inhibition	Vimseltinib	Reduces M2‐type TAMs, decreases immunosuppression [158, 159, 160, 161, 162]	Advanced tumors, tenosynovial giant cell tumor	NCT03069469	Phase 1/2	2024‐11‐20	Recruiting
			Advanced tumors, tenosynovial giant cell tumor	NCT05059262	Phase 3	2024‐2‐24	Recruiting
			Chronic graft‐versus‐host disease (cGVHD)	NCT06619561	Phase 2	2025‐4‐17	Recruiting
mTOR inhibition	Rapamycin	Reduces microglial hyperactivation [8, 256, 257]	AD, aging‐related cognitive decline	NCT05342519	Phase 2	2024‐8‐9	Not yet recruiting
			Amyotrophic lateral sclerosis (ALS)	NCT03359538	Phase 2	2024‐9‐19	Completed
			Mild cognitive impairment, Alzheimer's disease	NCT04200911	Phase 2	2025‐1‐8	Completed
			Alzheimer's disease and cognitive health	NCT04629495	Phase 2	2024‐08‐13	Recruiting
TGF‐β inhibition	Galunisertib (LY2157299)	Reduces fibrosis‐associated M2‐like polarization [258, 259, 260, 261, 262]	Liver fibrosis, tumor microenvironment	NCT02906397	Phase 1	2022‐3‐25	Completed
			Hepatocellular carcinoma (HCC)	NCT02178358	Phase 2	2022‐2‐25	Completed
			Hepatocellular carcinoma (HCC)	NCT01246986	Phase 2	2021‐1‐12	Completed
			Recurrent glioblastoma (GB)	NCT01582269	Phase 2	2024‐10‐23	Completed
			Newly diagnosed malignant glioma	NCT01220271	Phase 1/2	2017‐2‐16	Completed
			Locally advanced rectal cancer	NCT02688712	Phase 2	2025‐3‐10	Not recruiting
JAK–STAT inhibition	Upadacitinib/Abrocitinib/baricitinib	Suppresses proinflammatory M1 polarization [254, 255]	Prurigo nodularis	NCT06773403	Phase 4	2025‐1‐15	Recruiting
			Primary Sjögren's syndrome	NCT06862284	Phase 2	2025‐3‐11	Not yet recruiting
			Severe ulcerative colitis	NCT06838845	Observational	2025‐4‐23	Recruiting
			Moderate‐severe atopic dermatitis	NCT06684522	Phase 1	2024‐11‐12	Not yet recruiting
AhR agonism	Tapinarof (benvitimod)	Inhibits IL‐17 pathway, modulates M1/M2 balance [252, 253]	Pediatric plaque psoriasis	NCT05172726	Phase 3	2024‐12‐10	Recruiting
			Adult plaque psoriasis	NCT03983980	Phase 3	2022‐10‐13	Completed
			Pediatric/adult atopic dermatitis	NCT05014568	Phase 3	2024‐07‐08	Completed
			Cutaneous lupus erythematosus	NCT06661213	Phase 1	2024‐10‐28	Not yet recruiting
			Atopic dermatitis	NCT05142774	N/A	2024‐7‐8	Completed
ROCK inhibition	Fasudil/WP‐0512	Reduces fibrosis‐associated macrophage activation [263, 264, 265, 266]	Dementia with wandering behaviors	NCT04793659	N/A	2022‐7‐11	Completed
			Early Alzheimer's disease	NCT06362707	Phase 2	2024‐10‐22	Recruiting
			Amyotrophic lateral sclerosis (ALS)	NCT03792490	Phase 2	2023‐11‐30	Completed
			Amyotrophic lateral sclerosis (ALS)	NCT05218668	Phase 2a	2025‐4‐8	Not recruiting
			Parkinson's disease	NCT05931575	Phase 2	2023‐9‐26	Recruiting
Tgm2/NF‐κB inhibition	Cysteamine (RP103)	Blocks NF‐κB–SASP feedback loop, inhibits SASP secretion [267]	Major depressive disorder	NCT00715559	N/A	2017‐4‐7	Terminated
			Schizophrenia	NCT01139125	Phase 2	2014‐11‐20	Terminated
			Huntington's disease	NCT02101957	Phase 2/3	2014‐4‐2	Unknown status
			Neurological complications of cystinosis	NCT02012114	Phase 2	2022‐5‐10	Completed
			Nonalcoholic steatohepatitis (NASH)	NCT00799578	Phase 1/2	2014‐01‐31	Completed
			Nonalcoholic fatty liver disease (NAFLD) in Children	NCT01529268	Phase 2/3	2021‐6‐10	Completed
			Asthma	NCT03883984	Phase 1	2021‐5‐6	Completed
			Cystic fibrosis exacerbations	NCT03000348	Phase 2	2021‐4‐14	Completed

This table summarizes ongoing and completed clinical trials investigating macrophage‐targeted therapies. Data are grouped by shared mechanistic pathways to facilitate comparison across different therapeutic strategies.

Abbreviations: ISRCTN, International Standard Randomised Controlled Trial Number registry; NCT, National Clinical Trial identifier; TAMs, tumor‐associated macrophages; HSC, hematopoietic stem cells; SASP, senescence‐associated secretory phenotype; AhR, aryl hydrocarbon receptor; CSF‐1R, colony stimulating factor 1 receptor; JAK–STAT, Janus kinase‐signal transducer and activator of transcription; mTOR, mechanistic target of rapamycin; ROCK, Rho‐associated coiled‐coil containing protein kinase; TGF‐β, transforming growth factor beta; Tgm2, transglutaminase 2; NF‐κB, nuclear factor kappa‐light‐chain‐enhancer of activated B cells.

*Data source*: ClinicalTrials.gov registry.

In summary, the origin, polarization, and functional states of macrophages play critical roles in various diseases. Future therapeutic strategies combining immunomodulation, mechanical interventions, and precise targeting methods can optimize macrophage functions to promote tissue repair rather than pathological remodeling.

### 5.3 Senescent Microglia Exaggerate and Prolong the M1‐Like and M2‐Like Features

#### 5.3.1 Polarization Reprogramming of Microglia in Homeostasis and Disease States

Microglia originate from the mesoderm and are derived from erythromyeloid progenitors within the YS in a CSF‐1R‐dependent manner. Expression of the hematopoietic marker CD45 and adult macrophage/microglia markers CD11b, F4/80, and CX3CR1 can be detected in myeloid cells in the developing brain as early as E9.5. Furthermore, age accumulation and the loss of the blood–brain barrier lead to an increase in the migration of monocyte‐derived triggering receptor expressed on myeloid cells 2 (TREM2)‐expressing disease inflammatory macrophages to the brain [32, 33]. Although these cells have different genetic and transcriptomic profiles and serve different functions at sites of CNS injury, activated microglia and infiltrating MoMFs exhibit similar phenotypes and can generally be categorized as macrophages/microglia. Microglia settle in the CNS throughout their lifespan in a manner independent of BM‐derived monocytes, representing a distinct entity within the mononuclear phagocyte system [32]. Under homeostatic conditions, the microglial population has a long lifespan, sustained through slow local proliferation without input from peripheral cells, whereas under diseased conditions, microglia exhibit rapid clonal expansion capacity [34]. Extensive depletion of microglia throughout the entire brain of adult mice using selective CSF‐1R inhibitors results in complete repopulation with new microglia within 1 week of inhibitor withdrawal [268]. Survival of microglia critically depends on CSF‐1R signaling driven by CSF‐1 and IL‐34. CSF‐1R‐deficient mice exhibit complete microglial absence lifelong [268, 269, 270, 271]. In the brain, the expression of IL‐34 mRNA is much higher than that of CSF‐1 mRNA in early postnatal development and adulthood, consistent with the crucial role of IL‐34 in regulating microglial homeostasis. As resident macrophages of the CNS, microglia are implicated in the pathogenesis of many neurodegenerative and neuroinflammatory diseases [272, 273]. However, mice depleted of microglia do not exhibit behavioral or cognitive abnormalities, suggesting that microglia are not essential for these tasks [268].

#### 5.3.2 Polarization Reprogramming of Microglia in Homeostasis and Disease States

In CNS, when exposed to proinflammatory cytokines such as IFN‐γ, TNF‐α, and cellular or bacterial fragments, microglia utilize a range of immune receptors to recognize harmful stimuli and polarize toward a proinflammatory M1‐like phenotype [274, 275, 276]. These cells then produce proinflammatory cytokines (e.g., IL‐1α, IL‐1β, IL‐6, IL‐12, IL‐23, TNF‐α), chemokines, and MHC II, and express high levels of redox molecules (NADPH oxidase, phagocytic oxidase, iNOS). M1 macrophages also upregulate levels of TLR2, TLR4, Fc receptors (e.g., CD16, 32, 64), and chemokine receptor CCR7, expressing higher levels of costimulatory molecules CD80 and CD86, thus generating effective antigen presentation capabilities, aimed at killing invading pathogens and polarizing T cells to generate adaptive immune responses [274, 275, 277, 278, 279].

Following exposure to factors such as IL‐4 and IL‐10 produced endogenously by microglia or astrocytes, microglia are stimulated to polarize toward an M2‐like phenotype [280]. M2‐like polarization of primary microglia in vitro is similar to that of peripheral macrophages [281, 282], resolving inflammation by anti‐inflammatory mediators (IL‐10/IL‐13/TGF‐β), GFs (VEGF/EGF), and Arg1 [281, 283] to inhibit proinflammatory cell phenotypes, downregulate inflammatory cells, and mediate immune suppression, tissue remodeling, and repair to restore internal balance. IL‐10 induces downstream STAT family transcription programs, stimulating the generation of genes such as Il10, Tgfb1, the macrophage mannose receptor Mrc1, and Fizz1. Surface markers of M2 macrophages include the expression of CD23, scavenger receptors CD163 and CD204, mannose receptor CD206, and CD209 (DC‐SIGN) [284]. M2 macrophages stimulated by IL‐10 are more efficient at engulfing activated targets than M1 macrophages, but the phagocytic activity of microglia may be limited compared with that of blood‐derived macrophages [285]. During disease, microglia can assist in combating infections or suppressing tumor growth [286, 287, 288]. However, they may be less beneficial in autoimmune neuroinflammatory conditions such as multiple sclerosis [43, 289, 290] or amyotrophic lateral sclerosis (ALS) [291].

#### 5.3.3 Diseases and Chronic Inflammaging Cause Polarization Disorders of Microglia

The population of brain macrophages, including microglia and MoMFs, plays a pivotal role in the pathogenesis of aging and neurodegenerative diseases, such as Alzheimer's disease (AD) [292, 293]. During inflammatory stimuli, microglia isolated from aged mice exhibit excessive activation compared with young microglia [10]. Aged microglia display hyperactive proinflammatory responses, a phenomenon referred to as microglial priming [294, 295] (Figure 5). In contrast to the highly ramified, extensively branched morphology of microglia in a steady‐state healthy condition, the morphology of microglia in aged mice changes, with shorter and thicker processes and upregulation of IFN‐regulated genes or genes related to inflammation, phagocytosis, and lipid metabolism [296, 297]. Aged microglia show elevated proinflammatory cytokines and age‐dependent MHC II upregulation [8, 9, 10]. But, in a stable state, aged microglia are not inherently more inflammatory but rather exhibit downregulation of homeostatic and inhibitory genes (CD200, CX3CL1, CD47) and upregulation of stimulatory genes (MHC, CD86, CD68, CR3, and pattern recognition receptors such as TLRs and Clec7a) [32, 295, 298], predisposing them to mount stronger responses to stimuli in response to inflammation. However, the IL‐4Rα is impaired because of the decreased sensitivity of aged microglia to the anti‐inflammatory and M2‐promoting effects of IL‐4. The diminished IL‐4/IL‐4Rα response leads to reduced expression of arginase and CCL2 and decreased recruitment of IL‐4Rα^+^ macrophages, resulting in the compromised anti‐inflammatory function of aged microglia [299, 300].

Aged microglia also exhibit metabolic changes and downregulation of oxidative phosphorylation genes. The upregulation of mTOR signaling‐dependent translation and heightened protein levels of inflammatory mediators serve as a key driver in the microglia priming in aging and neurodegenerative conditions [8, 256, 257]. In aged microglia, mTOR signaling upregulates mTOR complex 1 signaling through the 4E‐PB1–eIF4E axis, leading to the translation of downstream mRNA and upregulation of NF‐κB‐dependent priming genes. However, decreased phosphorylation of 4EBP1 at the translational level results in reduced binding of eIF4E to eIF4G, leading to reduced levels of cytokine proteins, decreased microglial activation, and milder disease behaviors in mice [8]. Activation of mTOR signaling in microglia upregulates the TREM2 to activate the PI3K/AKT–mTOR and TREM2–APOE pathways [8, 301], which are essential for the microglial response to amyloid‐beta deposits responsible for inducing proinflammatory cytokines in microglia [302]. Pharmacological inhibition of this mTOR with rapamycin can reverse many age‐related features associated with HSC aging, thereby regulating the therapeutic regeneration of aged HSCs [257]. Studies utilizing scRNA‐seq of murine brain myeloid cells have demonstrated an accumulation of neuroprotective traits and monocyte‐derived TREM2 expressing disease‐associated inflammatory macrophages during aging [32]. TREM2 deficiency increases amyloid formation in early disease stages, followed by a decrease in amyloid accumulation with age [301, 303]. Mutations in TREM2 disrupt the energy state and function of microglia, affecting their ability to protect the brain from amyloid plaque deposition, which is associated with neurodegenerative diseases such as AD [43, 302, 304], multiple sclerosis [43, 289, 290], Parkinson's disease (PD) [305], and ALS [291]. The exaggeration and prolongation of this M1‐like and M2‐like feature in older microglia makes it harder for them to return to a state of internal equilibrium than in younger mice, and prolonged inflammation or exacerbation of aging contributes to cognitive impairments, excessive neuroinflammatory responses, diseases, and depression‐like behaviors, reaching levels commonly observed in neuroinflammatory environments [9, 10, 300].

#### 5.3.4 Regulation of Neuroinflammation in Inflammaging Brains

In contrast to microglia, aged MoMFs lack proinflammatory cytokines and are impaired in phagocytosis and chemotactic responses. Aging microglia exhibit metabolic dysfunction, enhanced proinflammatory phenotypes, and reduced phagocytic capacity in neurodegenerative diseases such as AD and PD, leading to persistent neuroinflammation and cognitive impairment. In recent years, therapeutic strategies targeting microglial polarization and function have demonstrated preclinical efficacy, primarily focusing on the clearance of senescent cells, modulation of their polarization, metabolic intervention, and exogenous supplementation.

For instance, MoMFs exhibit distinct aging phenotypes and origins compared with microglia, pharmacological mobilization of monocytes from the BM can enhance the function of aged circulating monocytes and reverse HSCs aging, thereby repairing damaged or aging neurons [72, 73]. In AD or mouse demyelination models, drugs such as CSF‐1 can promote the mobilization and migration of BM‐derived macrophages to the CNS, facilitating remyelination within lesions, or increasing the macrophage presence in the CNS [292, 306, 307]. Microglial autophagy protects against amyloid plaque accumulation and aging. Defects in autophagy promote microglial senescence both in vivo and in vitro, and autophagic dysfunction is one of the pathogenic mechanisms of AD [308]. Targeting the dysregulation of autophagy in microglia, current senolytic therapies aim to remove senescent microglia to achieve neuroprotection. For example, the senolytic combination of dasatinib and quercetin has been shown to reduce amyloid plaque burden and improve cognitive function in AD mouse models [308].

Similarly, targeting senescent microglial clearance (senolytic therapy) includes inhibitors of Tgm2, such as cysteamine. Cysteamine inhibits the NF‐κB–SASP positive feedback loop, alleviating neurodegenerative phenotypes in aged mice [267]. Oral administration of cysteamine significantly improved age‐related neuropathological phenotypes in elderly mice, suggesting that cysteamine may serve as a novel suppressor of neurodegeneration‐related phenotypes [267]. Perhaps gene editing technologies (such as CRISPR targeting TREM2 or TGM2) can be utilized to precisely regulate the function of microglia, and nanocarriers can be used to deliver anti‐inflammatory drugs to reduce systemic side effects.

Metabolically, aged dura mater exhibits lymphatic dysfunction due to TGF‐β1‐driven extracellular matrix (ECM) remodeling and perilymphatic collagen deposition, impairing CNS waste clearance. This finding suggests that targeting dural immune cell‐mediated ECM remodeling and fibroblasts could serve as a potential therapeutic strategy for restoring waste clearance in the aging brain [309]. Galunisertib (LY2157299) is a selective inhibitor of TGF‐βRI. It blocks TGF‐β signaling by directly inhibiting the kinase activity of TGF‐βRI. This inhibitory effect can reduce the downstream signals mediated by TGF‐β, including phosphorylation of Smad proteins, thereby influencing processes such as cell proliferation, differentiation, apoptosis, and ECM production. For example, galunisertib plays a crucial role in the treatment of malignant gliomas, including newly diagnosed and recurrent GBM, where TGF‐β promotes tumor cell survival, invasion, and immune evasion [258]. Similar to it, LY2109761 dually inhibits TGF‐β receptor type I and type II (TGF‐βRI/II) kinases. Its mechanism of action is to simultaneously inhibit the kinase activities of TGF‐βRI and TGF‐βRII, thereby more comprehensively blocking TGF‐β signal transduction. LY2109761 has shown strong inhibitory effects on TGF‐β‐induced cellular responses in experiments, enhancing the radiation response of GBM and prolonging survival, and is also considered a potential antifibrotic and anticancer drug [259]. Moreover, in liver diseases, galunisertib demonstrates therapeutic potential by reducing M2‐like polarization, which is closely associated with profibrotic remodeling. In hepatocellular carcinoma (HCC), where chronic inflammation and fibrosis contribute to a tumor‐permissive microenvironment, galunisertib exerts antitumor effects through multiple mechanisms. By inhibiting TGF‐β1‐mediated signaling, it suppresses the stemness phenotype of HCC cells via modulation of CD44 expression, inhibits the activation of hepatic stellate cells, and shifts macrophage polarization from a profibrotic M2‐like state toward a more balanced or reparative phenotype. This results in reduced ECM deposition and improved liver function. Galunisertib is under clinical investigation for the treatment of advanced or recurrent HCC, both as monotherapy and in combination with other targeted agents or immunotherapies [260, 261]. Apart from brain and liver diseases, galunisertib also plays a role in the treatment of other cancers. It is currently under research in the combined application of neoadjuvant radiotherapy and chemotherapy for locally advanced rectal cancer [262].

### 5.4 The Critical Role of M1/M2 Balance in Kupffer Cells during Liver Pathology

#### 5.4.1 Kupffer Cells of Different Origins Exhibit Ontogeny‐Dependent Functional Specialization

Similar to the approach used to discuss Langerhans cells in the skin and microglia in the brain, we will explore the largest population of resident tissue macrophages in the liver—Kupffer cells—in the order of their multiorigin, disease‐associated multidirectional polarization, and functional changes with aging that contribute to the occurrence and progression of liver pathology. As the liver's dominant resident macrophages, Kupffer cells within hepatic sinusoids clear senescent cells, virus‐infected cells, immune complexes, and immunostimulatory microbial products from intestinal translocation to prevent liver damage and systemic immune reactions [310, 311, 312]. Emerging evidence indicates that the dynamic balance between proinflammatory (M1) and anti‐inflammatory (M2) Kupffer cell subsets is crucial for maintaining liver homeostasis and influencing disease progression. The majority of Kupffer cells are believed to originate from primitive hematopoiesis before the appearance of HSCs or monocyte precursors [38, 39]. The final hematopoiesis derived from HSCs is retained in the FL of mice until approximately embryonic day 18 or 12 weeks after human conception, after which it migrates to the BM and persists into adulthood [23]. Macrophages derived from HSCs differentiate from BM‐circulating myeloid progenitor cells via a process mediated by macrophage CSF‐1. In CSF‐1‐deficient mice, the number of Kupffer cells decreases and exhibits immature morphology, while CSF‐1 administration leads to immediate proliferation and maturation of Kupffer cells, highlighting the crucial role of CSF‐1 in Kupffer cell differentiation and proliferation [23]. After the engraftment of BM‐derived circulating monocytes into the liver, they can promote self‐renewing and fully differentiated Kupffer cells [38, 40]. While monocyte‐derived Kupffer cells and embryonically derived Kupffer cells share similar functions and morphology, at least 12 genes, including CD163 and CCR3, show differential expression [38]. In an inflammatory state, both resident and MoMFs are involved in migration, expansion, and signal transduction [25, 41], infiltrating macrophages in the mouse liver are derived from BM‐derived Ly6C^hi^ monocytes [26, 56]. Early on, classical proinflammatory (CCR2^hi^ CX3CR1^low^) monocytes are driven by chemotactic proteins produced by monocytes, Kupffer cells, stellate cells, or hepatocytes, recruited to the site of inflammation and persist for at least 48 h, forming a ring‐like structure around the injured area. Subsequently, under the influence of local cytokines, they undergo reprograming to become nonclassical or alternative monocytes, transitioning from CCR2^hi^ CX3CR1^low^ to CX3CR1^hi^ CCR2^low^, and then entering the injured site to promote proper wound healing, known as the CCR2–CCL2 interaction [27]. In addition, monocytes are recruited to the liver through pathways such as the CXCR3–CXCL9 [74], CXCR3–CXCL10, CCR1–CCL5, and CCR8–CCL1 axes [75, 76, 313], and extravasate into the liver and differentiate into Kupffer cells [23, 39] to play their role in liver function. These findings underscore that Kupffer cell heterogeneity—shaped by origin and microenvironment—dictates their functional specialization in liver homeostasis and injury responses.

#### 5.4.2 M1/M2 Imbalance in Liver Disease

Kupffer cell plasticity—manifested as M1‐like/M2‐like polarization—dynamically regulates liver pathology. M1‐like polarization drives hepatocyte dysfunction through proinflammatory factor secretion (e.g., ROS, chemokines), exacerbating injury and carcinogenesis [312, 314]. Intriguingly, in murine models, adoptive transfer of M1‐like macrophages more effectively reduces liver fibrosis than M0 macrophages, likely by inducing hepatic stellate cell apoptosis and recruiting endogenous immune cells [315], Conversely, M2‐like Kupffer cells exhibit minimal antifibrotic activity [315], highlighting polarization‐dependent therapeutic outcomes.

However, chronic inflammation tilts the balance toward M2‐like dominance. With the persistence of chronic inflammation, other cytokines, such as IL‐4 and IL‐13, promote the activation of Kupffer cells into the anti‐inflammatory M2‐like subgroup, leading to the secretion of anti‐inflammatory cytokines, which induce wound repair and fibrosis [25, 41, 314, 316]. This sustains hepatic immune tolerance and via anti‐inflammatory niche maintenance. In particular, IL‐10, as an anti‐inflammatory cytokine, can downregulate the effector functions of macrophages and the differentiation of adjacent cells, maintaining the steady state of the immune microenvironment. Mechanistically, M2‐like Kupffer cells, by secreting IL‐10, activate high‐inducible NO synthase in M1‐like Kupffer cells, promoting apoptosis of M1‐like Kupffer cells and maintaining the homeostasis and balance of M1/M2 Kupffer cell populations [317].

In cancer, the M1/M2 ratio is subverted to favor immune evasion [82, 83]. Tumor cells utilize the TRIM65–JAK1/STAT1 axis to suppress M1‐like polarization of macrophages and promote tumor growth. Inhibiting the JAK1/STAT1 signaling pathway by knocking out TRIM65 polarizes tumor‐associated macrophages to the M1‐like phenotype, thus inhibiting the development of HCC [83], while gut microbiota‐derived d‐lactate reprograms M2‐like tumor‐associated macrophages toward an M1‐like phenotype via PI3Kδ‐dependent lactate sensing, enhancing the ability of Kupffer cells to clear pathogens and reshape the immunosuppressive TME in HCC mice [82]. Although the expression levels of MHC II molecules in Kupffer cells are lower than those in traditional DCs, Kupffer cells can promote immune tolerance by secreting anti‐inflammatory factors, including IL‐10, TGF‐β, and prostaglandin E2, inducing regulatory T cells, a function that traditional DCs do not have [318, 319, 320]. This mechanism that affects the degree of damage to the body and the outcome of the disease by altering the polarization of Kupffer cells also provides us with new ideas for achieving therapeutic effects by regulating its phenotype.

#### 5.4.3 Kupffer Cell Dysfunction in Inflammaging

At the same time, it is inevitable that when the body is in a nonhomeostasis state, such as the inflammaging and cancer we have mentioned many times, the biology of Kupffer cells will be reshaped, exacerbating inflammatory responses while impairing tissue regeneration. Age‐related metabolic dysfunction further skews Kupffer cells toward M1‐like polarization. A decline in liver structure and cell function is observed with age, disrupting sinusoidal homeostasis, increasing the number of polyploid, damaged, and aging hepatocytes, and impairing the function of liver cells and macrophages, leading to reduced albumin production or exogenous metabolism [15, 16]. Aging liver sinusoidal endothelial cells secrete proinflammatory cytokines, increase SASP, oxidative stress, and ROS, exhibit high expression of alpha‐smooth muscle actin, and accumulate large numbers of lipid droplets internally [15, 16]. The proinflammatory environment, expression of adhesion molecules in liver sinusoidal endothelial cells, and decreased liver blood flow promote the recruitment of inflammatory cells, including Kupffer cells and neutrophils, which secrete more proinflammatory cytokines, sustaining the proinflammatory state of the aging liver [15, 321, 322] (Figure 5). As individuals age, an increasing number of preadipocytes differentiate into mature adipocytes, aging does not directly promote hepatic steatosis but increases liver cell damage and inflammation, where the enhanced M1 macrophage polarization likely plays a significant role [323]. Cytotoxic T cells and macrophages gradually infiltrate into obese adipose tissue, dysfunctional lipid metabolism can lead to dysfunction of macrophages, which has been summarized in other reviews, so it will not be repeated [324]. T cell‐derived cytokines, such as IFN‐γ, promote the recruitment and activation of M1 macrophages, thereby enhancing adipose tissue inflammation and insulin resistance. Activated macrophages transform into M1 macrophages, releasing cytokines such as IL‐1β, IL‐6, and TNF‐α [325, 326], while factors associated with M2 macrophage polarization, such as adiponectin secretion, decrease correspondingly. This generates a chronic low‐grade inflammatory milieu that promotes atherosclerosis and other diseases [327].

Autophagy dysregulation underlies age‐associated dysfunction. Autophagy regulation can prevent excessive inflammation and maintain macrophage function during the aging process, improving immune responses and reducing the incidence and mortality associated with inflammatory aging [328]. With age, autophagy, autophagic clearance of damaged components, is dysregulated in the macrophage population [328]. The loss of the autophagy genes ATG5 and ATG7 in BM‐derived macrophages results in decreased antigen presentation capability, impaired maturation, altered mitochondrial metabolism, downregulation of surface receptors such as TLR4, and increased secretion of proinflammatory cytokines [328, 329, 330, 331]. Aging also promotes the formation of the NLR family pyrin domain‐containing 3 (NLRP3) inflammasome mediated by the stimulator of interferon genes signaling pathway in macrophages. This serves to regulate macrophage proinflammatory activation, leading to the production of proinflammatory cytokines such as IL‐18 and IL‐1β, and exacerbating liver ischemia–reperfusion injury [313, 332]. This can be mitigated by knocking down NLRP3 in macrophages or inhibiting stimulator of interferon genes to suppress the excessive secretion of proinflammatory cytokines/chemokines, thereby reducing IR injury in elderly patients [313]. Although NLRP3 may confer protection against hepatitis C virus and hepatitis B virus infections [332].

Although an increase in the number and basal activity of Kupffer cells with age has been observed in rat models and in the livers of elderly individuals without pathological lesions [333], the activation capacity of Kupffer cells is impaired in the aging macrophage population, leading to a weakening of extracellular pathogen phagocytosis and antigen presentation [40]. This might be caused by the continuous chronic activation of Kupffer cells in the liver of aged rats, resulting in a decreased response to stimuli. This is specifically reflected in the fact that aging rats respond less to Kupffer cells than young rats. For example, under the same degree of hepatotoxic stimulation (such as cadmium), Kupffer cells are only activated in young rats and not activated in old rats, which is very important for inflammatory liver injury [334]. Fortunately, though this decrease in phagocytic ability is driven by age‐related changes in the local microenvironment, it is reversible, and no significant differences in phagocytic ability due to aging were found in BM‐derived macrophages [40].

Given the reversibility of age‐related functional decline in Kupffer cells, a potential therapeutic strategy is to replenish or rejuvenate the liver's immune clearance capacity by introducing exogenous macrophages, such as BM‐ or blood‐derived macrophages [335, 336]. For instance, autologous macrophage therapy has shown promise in liver fibrosis research: in liver regeneration models, MoMFs have been shown to dynamically regulate inflammation and repair, clear necrotic tissue, and promote hepatocyte regeneration [337]. M2‐like macrophages play a hepatoprotective role in acute‐on‐chronic liver failure by suppressing the necroptosis–S100A9–necroinflammation axis, suggesting that modulating macrophage polarization can improve liver function [335]. Additionally, metabolic interventions based on metabolic reprogramming provide further support for macrophage‐based therapies in liver diseases. Targeting ferroptosis in macrophages (e.g., by inhibiting the NCF1–TLR4–hepcidin axis) can improve iron homeostasis in Kupffer cells, thereby slowing the progression of metabolic‐associated steatohepatitis [338]. Exogenous itaconate enhances the ability of macrophages to phagocytose apoptotic cells by activating the Nrf2‐TIM4 pathway, promoting their conversion to M2‐like macrophages and alleviating autoimmune liver injury [336]. However, it is important to note that while these studies support the feasibility of restoring aged Kupffer cells function through cell therapy or metabolic interventions, the efficacy and safety of these approaches still require further clinical validation. Future research should focus on identifying the optimal conditions for these treatments and evaluating their long‐term effects to ensure they can be safely and effectively applied in the clinical treatment of liver diseases.

### 5.5 Age‐Related Changes in Skeletal Muscle Macrophages

#### 5.5.1 Dynamic Polarization Transition of Macrophages during Muscle Repair

Unlike other tissue‐resident macrophages, skeletal muscle macrophages demonstrate niche‐specific plasticity during repair. Recent advances in single‐cell sequencing and spatial transcriptomics have illuminated the dynamic polarization of skeletal muscle macrophages, revealing their roles in proinflammatory and aging characteristics of muscle [339, 340, 341, 342, 343]. A comparison of the transcriptome profiles of infiltrating myeloid cells from healthy, acutely injured, and early dystrophic muscles showed that the largest components of the infiltrating myeloid group were BM derived, the most prominent of which were monocytes and macrophages, and the continuous emergence of specific macrophage subtypes coordinated skeletal muscle regeneration [342]. In skeletal muscle, resident macrophages are primarily present in the interstitial tissue in the form of CD45^+^F4/80^+^CD64^+^Ly6C^low^ cells. These cells express tissue‐specific transcription factors and display muscle‐specific functions under steady‐state conditions [344]. Studies have shown that resident macrophages from different types of skeletal muscles, such as the quadriceps and diaphragm, exhibit highly similar gene expression profiles but are significantly distinct from macrophages in the peritoneum and alveoli [344]. Notably, resident macrophages of skeletal muscle show higher expression levels of genes related to immune responses as well as those promoting skeletal muscle growth and regeneration. This characteristic may be attributed to the constant mechanical stress endured by skeletal muscles. Due to the high frequency of tissue damage and microenvironmental changes caused by mechanical stress, these macrophages are more actively involved in maintaining tissue homeostasis, clearing debris, and supporting muscle repair and regeneration [344].

During acute muscle repair, both resident and infiltrating macrophages orchestrate regeneration [342]. Circulating monocytes migrate to injury site and undergo in situ phenotype into reparative macrophages—independent of continuous monocyte recruitment [345, 346]. Injured skeletal muscle recruits LY6C^hi^CCR2^+^ CX3CR1^low^ monocytes via CCR2 chemotaxis signaling [347, 348], produces high levels of insulin‐like growth factor‐1 (IGF1) to promote muscle regeneration [348], play a key role in regulating muscle tissue repair by modulating satellite cells to restore tissue integrity and function, clearing debris, and secreting growth/differentiation factors such as GDF15, GDF3, IGF1, other GFs, and ECM proteins [341, 349]. During the early phase, they produce high levels of proinflammatory mediators to propagate inflammation [263, 350]. As repair progresses, these macrophages undergo polarization shifts—from a proinflammatory (M1‐like) state to an anti‐inflammatory [342, 351], proregenerative (M2‐like) phenotype—critical for resolving inflammation and supporting tissue regeneration. During the initial inflammatory phase, M1 macrophages primarily participate in the early inflammatory response and induce apoptosis of fibro/adipogenic progenitors via TNF, thereby preventing excessive fibrosis [351] [352]. In contrast, during the later stages, M2 macrophages support tissue reconstruction by secreting anti‐inflammatory mediators to resolve inflammation [350], promoting fibro‐adipogenic progenitors (FAPs) proliferation, and driving active resolution of inflammation and expression of GFs, which aid in either fibrosis or regeneration [339, 340, 341, 353]. This process is crucial for the treatment of muscular dystrophy [350, 351]. Observations from ischemia–reperfusion injury models reveal that promoting M1‐like polarization and inhibiting M2‐like polarization both impede myoblast differentiation [354]. However, activation of the cannabinoid receptor 2 can partially protect skeletal muscle from such injuries via the Nrf2 signaling pathway [354, 355]. Single‐cell analysis of mouse muscle also shows that macrophages in the quadriceps highly express M2‐like genes (such as MRC1 and FCGRT), indicating their enhanced tissue repair capabilities. This dominance of M2‐like polarization may help maintain muscle homeostasis and counteract aging‐related changes in the muscle microenvironment and chronic injuries [344].

Single‐cell transcriptomic analyses of acutely injured muscle reveal that macrophages exhibit multiple subtypes over time, including proinflammatory (M1), IL7R+, complement gene‐enriched, and antigen presentation‐associated subtypes. The proportions of these subtypes dynamically change to meet the demands of different repair phases [342, 356]. Time‐specific gene expression analyses show that the direct response to muscle injury is initially controlled by proinflammatory phenotypes, which later transition to anti‐inflammatory phenotypes [342, 351, 356]. This dynamic polarization process is particularly relevant to aging, as macrophages regulate tissue regeneration mainly by altering their phenotypes and functions [354]. Impaired plasticity can delay repair, highlighting the importance of understanding macrophage behavior in age‐related muscle dysfunction.

#### 5.5.2 Aging Alters Macrophage Polarization Dynamics

In aging muscle, the balance between M1 and M2 macrophage polarization is disrupted. Comparative analysis of young and aged skeletal muscle macrophage subsets reveals significant enrichment of genes associated with aging (e.g., Spp1, Fabp5) and inflammation (e.g., Fabp4, Il1b, S100a8, and S100a9) in aged skeletal muscle macrophages, indicating chronic inflammation and aging characteristics [263, 343, 357, 358]. In contrast, these genes are expressed at lower levels in young skeletal muscle macrophages. Studies suggest that during asynchronous chronic inflammation, macrophage polarization shifts toward a proinflammatory M1‐like phenotype rather than an anti‐inflammatory and reparative M2‐like phenotype, potentially disrupting the muscle microenvironment balance and inhibiting regeneration [21, 22]. Additionally, the increased expression of antioxidant enzyme mRNAs reflects an adaptive response to elevated ROS, further suggesting that the shift in macrophage polarization toward a proinflammatory and aging phenotype may impair the regenerative capacity of aged muscle [343, 359]. In addition, reduced neutrophils and monocyte/macrophage chemokines produced by macrophages of skeletal muscle in old age may also lead to delayed damage repair [343].

Satellite cells, crucial for muscle growth and repair, are affected by aging. Studies suggest young environments enhance old satellite cell proliferation, improving regeneration, while aged environments impair young muscle repair, highlighting the role of macrophages in this process [360, 361]. Transplanting young BM cells into aged recipients can prevent sarcopenia and protect against age‐related changes in muscle fiber phenotype. Conversely, transplanting old BM cells into young animals can reduce satellite cell numbers and promote their transition to a fibrotic phenotype [362, 363]. Notably, aging‐related changes in the muscle microenvironment further drive abnormal macrophage activation. Dysregulated inflammatory processes during aging, combined with BM cell senescence, lead to an age‐related decline in satellite cell number and function. This disrupts central mechanisms that regulate skeletal muscle morphology and remodeling through inflammation‐induced changes, leading to reduced regenerative capacity, poor perfusion, oxidative stress, mitochondrial dysfunction, and chronic inflammation [339]. These factors drive the muscle changes associated with a frailty phenotype, ultimately resulting in more degenerative and dysfunctional skeletal muscle [339, 340, 364]. Aging BM cells increase the transition of satellite cells toward a fibrotic phenotype, suggesting that immune system aging may indirectly impair muscle repair and regeneration by altering macrophage polarization states. This exacerbates age‐related muscle fibrosis [24]. Pharmacological interventions targeting relevant pathways, such as specific androgen receptor modulators, could be used to treat frailty phenotypes and age‐related chronic diseases [340].

Although M2‐like polarization can promote tissue homeostasis during the repair phase, in chronic inflammatory conditions such as aging, prolonged M2‐like polarization can drive fibrosis. Wang et al. [363] demonstrated in a mouse model that anti‐inflammatory M2a macrophages increase in aged muscle, contributing to muscle fibrosis. This reparative phenotype of macrophages is prematurely activated, impairing the clearance of damaged tissue. They further found that M2a macrophages and related muscle fibrosis in aged muscle are partially determined by the age of BM cells [363] (Figure 5). This finding aligns with the view that the function and fate of tissue macrophages are determined by their hematopoietic origin [47]. In the mdx mouse model, macrophages also tend to polarize toward the M2‐like phenotype, highly expressing TGF‐β1, which prevents the apoptosis of FAPs and promotes their differentiation into matrix‐producing cells, thereby exacerbating muscle fibrosis [352, 365]. Nilotinib reduces fibrosis by blocking TGF‐β1 [352]. These findings reveal the role of TNF in controlling fibrosis and highlight the importance of the transition from a TNF‐rich environment to a TGF‐β‐rich environment in preventing the worsening of fibrosis during aging. In muscular dystrophy models, macrophage‐specific deletion of Nfix delays fibrosis progression through mechanisms involving increased TNF‐α and decreased TGF‐β1, restoring FAPs apoptosis [263]. Meanwhile, single‐cell and spatially resolved transcriptomic studies validated by immunofluorescence have also confirmed that, in the TME (e.g., GBM), GPNMB+ macrophages competitively inhibit the antigen‐presenting function of DCs, thereby impeding T‐cell activation [344, 366]. A similar mechanism may exist in chronic muscle inflammation: M2‐like macrophages that accumulate in aging or injured muscle may suppress effector T‐cell function by expressing immune inhibitory molecules such as GPNMB or PD‐L1, thus hindering regeneration. Targeting these macrophage subsets (e.g., using anti‐GPNMB antibodies or CXCR3/CXCL10 pathway agonists) could help restore a proreparative immune microenvironment. Additionally, promoting proinflammatory macrophages (e.g., by inducing CXCL9/CXCL10 secretion via IFN‐γ) can enhance T‐cell recruitment, synergistically promoting muscle regeneration [344].

#### 5.5.3 Therapeutic Strategies for Rebalancing Macrophage Polarization

Macrophage polarization dynamically regulates muscle repair: M1‐like macrophages mitigate fibrosis via TNF‐mediated FAP apoptosis acutely, whereas sustained M2‐like polarization drives TGFB1‐dependent fibrogenesis chronically. Based on this mechanism, future therapies could consider combining TGF‐β1 inhibitors with proinflammatory factors, such as nilotinib in conjunction with TNF‐α agonists, to balance macrophage polarization and restore normal FAPs apoptosis. Alternatively, targeting Nfix or the Rho‐associated coiled‐coil containing protein kinase (ROCK) pathway by developing macrophage‐specific siRNA or small‐molecule drugs could inhibit profibrotic macrophage subsets. Studies have shown that ROCK inhibitors, such as fasudil and WP‐0512, exert anti‐inflammatory and cytoprotective effects through modulation of macrophage polarization. However, recent findings reveal that ROCK inhibition may paradoxically enhance the profibrotic activity of M2‐polarized macrophages by upregulating Nfix, this discovery highlights the dual nature of ROCK inhibition: while it can effectively reduce inflammation, it may also exacerbate tissue fibrosis under certain conditions [263]. Based on this understanding, direct targeting of Nfix has emerged as a promising new strategy for optimizing macrophage regulation. By specifically inhibiting Nfix expression, the profibrotic phenotype of M2 macrophages can be reversed, demonstrating significant antifibrotic effects in disease models such as muscular dystrophy [264, 265]. Notably, WP‐0512, a next‐generation ROCK inhibitor currently in a Phase IIa clinical trial for ALS (NCT05218668), may offer improved specificity in modulating macrophage polarization compared with fasudil, suggesting enhanced therapeutic potential in fibrotic diseases (Table 1). In the context of neurological disorders, particularly AD, abnormal activation of the ROCK–Nfix pathway has been linked to imbalanced microglial (brain‐resident macrophage) polarization. Fasudil has been shown to restore the balance between M1‐like and M2‐like microglial phenotypes, enhance cerebral perfusion while suppressing neuroinflammation [264, 266]. Combining ROCK and Nfix inhibition may produce synergistic effects by reshaping macrophage/microglial function, thereby protecting neural circuits and slowing cognitive decline. These research advances provide a crucial theoretical foundation for the development of novel antifibrotic therapies based on macrophage polarization regulation, especially in the treatment of muscle disorders and neurodegenerative diseases. Another approach may involve blocking the immunosuppressive function of GPNMB+ macrophages in muscle or infusing ex vivo polarized proreparative macrophages (e.g., M1‐like or transitional macrophages) to remodel the injury microenvironment.

Although targeting macrophage polarization demonstrate translational potential for treating muscle diseases, this process is highly dynamic in vivo. Macrophages in different polarization states play distinct roles at various stages of injury, and current technologies are not yet able to precisely control the timing of interventions to modulate their polarization. Our current understanding of macrophage subsets in muscle primarily relies on single‐cell atlases, but these maps still need further refinement to identify more specific targets. Investigating the mechanisms of these transcription factors in greater depth, particularly through cell‐type‐specific knockout experiments, will help us better understand how macrophage polarization is regulated during aging. By unraveling the molecular mechanisms of macrophage polarization and developing targeted intervention strategies, we may provide groundbreaking treatments for muscle aging and related degenerative diseases, ultimately achieving the restoration and long‐term maintenance of muscle function.

## 6 New Macrophage Therapy: From Polarization Regulation to Cell Engineering

In addition to traditional therapeutic strategies targeting macrophage polarization or aging‐related signaling pathways—such as TGF‐β inhibitors, AhR agonists, and JAK–STAT inhibitors—novel cell‐based therapies have recently emerged with revolutionary potential in the field of macrophage modulation. These approaches go beyond small‐molecule interventions on signaling pathways and instead directly modify or replace macrophages themselves, enabling more precise immune regulation.

### 6.1 CAR‐Engineered Macrophages

Senolytic therapy selectively eliminates proinflammatory senescent cells, autologous macrophage infusion replenishes functional immune cell populations, and chimeric antigen receptor macrophage (CAR‐M) therapy endows macrophages with tumor‐targeting capabilities through genetic engineering. These innovative strategies not only expand the therapeutic boundaries of macrophage‐based treatments, but also offer new hope for diseases that currently lack effective therapies, such as solid tumors, fibrosis, and neurodegenerative disorders.

In previous studies, CAR‐T/NK cell therapies have shown significant clinical efficacy in patients with relapsed or refractory hematologic malignancies. However, due to the extensive inhibitory pathways within the TME, which limit T‐cell activation, CAR‐T cells face particular challenges when entering dense tumor tissues, making CAR‐T therapy less effective for solid tumors. Leveraging the antigen‐presenting capabilities, potent tumor phagocytosis, and efficient trafficking to the TME of macrophages, CAR‐M represent a novel approach [367]. These modified macrophages can specifically recognize and phagocytose tumor cells, usually through adenovirus transduction to induce a stable M1‐type proinflammatory phenotype, characterized by high expression of inflammatory‐related genes. Activated M1 macrophages release proinflammatory cytokines such as IL‐1β, IL‐6, IL‐12, IL‐18, and TNF, forming a proinflammatory microenvironment and activating T‐cell responses, indicating the presence of systemic and collaborative immune functions [368, 369].

CAR‐M therapy, achieved through genetic editing, enables macrophages to specifically recognize and engulf tumor cells while reshaping the immune microenvironment, potentially overcoming major challenges associated with CAR‐T/NK therapies [370, 371]. Extensive experiments demonstrate that CAR‐M therapy possesses the capability to eliminate tumor cells both in vitro and in preclinical in vivo models. LPS‐activated macrophages secrete TNF, NO, and ROS—key effector molecules in CAR‐M's antitumor activity—which act synergistically [370, 371].

The extensive interaction between macrophages and other immune components plays a crucial role in tumor clearance. The researchers observed that CAR‐M not only can directly eliminate tumor cells, but also can act as antigen‐presenting cells to present tumor antigens to T cells, thereby activating T‐cell‐mediated antitumor immune responses and reshaping the TME [372, 373]. Moreover, by secreting cytokines such as CXCL8, CXCL9, CXCL10, CXCL11, and CCL57, it can increase the infiltration of antitumor neutrophils, CD8+ T cells, cytotoxic T lymphocytes and NK cells within the tumor [374].

Among the various mouse models of solid tumors (pancreatic cancer [375], breast cancer [370], HCC [376], and ovarian cancer [374]), CAR‐M has demonstrated remarkable potential in all of them. This highlights the potential of CAR‐M in enhancing antitumor immunity and improving the outcomes of solid tumors. A ongoing Phase I clinical trial (NCT04660929) is currently evaluating the safety and feasibility of CAR‐M therapy in HER2‐positive solid tumors [377] (Table 2).

**TABLE 2:** Novel Macrophage‐Targeted Therapeutic Strategies.

Strategy/approach	Mechanism	Disease application	Trial ID	Phase	Last update date	Status
**CAR‐macrophages**	**Macrophage infusion therapy**					
HER2‐targeted CAR‐M	Engineered macrophage phagocytosis [370, 374, 375, 376, 377]	HER2+ gastric cancer with peritoneal metastases	NCT06224738	Phase 1	2024‐01‐25	Not recruiting
		HER2+ solid tumors	NCT04660929	Phase 1	2024‐12‐18	Not recruiting
		Breast cancer organoids	NCT05007379	Observational	2021‐08‐16	Unknown status
**Senolytic therapy**	**Senescent cell clearance**					
Dasatinib + quercetin (D+Q)	BCL‐2/PI3K‐AKT inhibition [378]	Alzheimer's disease (AD) models	NCT04063124	Phase 1	2023‐03‐06	Completed
		Idiopathic pulmonary fibrosis	NCT02874989	Phase 1	2020‐05‐12	Completed
		Fibrotic Nonalcoholic fatty liver disease (NAFLD)	NCT05506488	Phase 1/2	2023‐02‐28	Recruiting
		Elderly obesity	NCT05653258	Phase 2/3	2025‐03‐24	Recruiting
		Diabetic nephropathy	NCT02848131	Phase 2	2024‐04‐19	Enrolling by invitation
UBX1325	BCL‐xL inhibition	Diabetic eye disease	NCT04537884	Phase 1	2022‐03‐10	Completed
Navitoclax (ABT‐263)	BCL‐2 inhibition [379]	Advanced solid tumors	NCT02079740	Phase 1/2	2025‐04‐24	Not recruiting
		High‐grade serous/Triple‐Negative Breast Cancer	NCT05358639	Phase 1	2024‐11‐18	Not recruiting
Fisetin	Inhibit NF‐κB signaling [380]	Elderly bone health	NCT04313634	Phase 2	2024‐07‐22	Completed
		Vascular function in aging	NCT06133634	Phase 1/2	2025‐03‐27	Recruiting
		Post‐COVID complications	NCT04476953	Phase 2	2025‐02‐17	Not recruiting
**Macrophage infusion**	**Functional macrophage replenishment**					
Autologous macrophage therapy	Functional macrophage replenishment [389, 392, 393, 394]	Acute liver injury	ISRCTN12637839	Phase 1	2025‐03‐11	Recruiting
		Liver cirrhosis	ISRCTN10368050	Phase 2	2025‐01‐14	Completed
Genetically engineered macrophage‐based cell therapy	Macrophages derived from iPSCs obtained through gene editing technology [396, 397]	Ovarian cancer (xenograft)	N/A	Preclinical	N/A	[396]
		Peritoneal gastric mets (murine)	N/A	Preclinical	N/A	[397]

This table summarizes current advances in genetically engineered macrophages (CAR‐M), senolytic therapies, and macrophage infusion strategies across oncology, fibrosis, and degenerative diseases.

Abbreviations: ISRCTN, International Standard Randomised Controlled Trial Number registry; NCT, National Clinical Trial identifier; CAR‐M, chimeric antigen receptor macrophage; PI3K, phosphoinositide 3‐kinase; JAK–STAT, Janus kinase‐signal transducer and activator of transcription; NF‐κB, nuclear factor kappa‐light‐chain‐enhancer of activated B cells; iPSCs, induced pluripotent stem cells; D+Q, dasatinib plus quercetin.

*Data sources*: ClinicalTrials.gov registry and ISRCTN registry.

### 6.2 Senolytic Therapy

As previously discussed, senescent cells accumulate during chronic inflammation and secrete SASPs, which promotes the polarization of macrophages toward an M2‐like phenotype. These M2‐polarized macrophages further release fibrotic factors, forming a positive feedback loop that exacerbates tissue damage. This phenotypic shift is particularly evident in fibrotic diseases such as pulmonary and liver fibrosis, as well as in neurodegenerative disorders like AD. Currently, major senolytic drugs exert their effects on macrophage function through distinct molecular pathways. Dasatinib + quercetin (D+Q) is a well‐established combination that targets the BCL‐2 and PI3K/AKT signaling pathways, effectively eliminating senescent cells [378]. Navitoclax (ABT‐263), a selective inhibitor of BCL‐2 and BCL‐xL, is particularly effective in clearing senescent HSCs [379]. Fisetin, a natural flavonoid, exhibits potent senolytic activity against senescent adipocytes [380]. These agents, while differing in their cellular and molecular selectivity, collectively contribute to the modulation of macrophage polarization and inflammatory responses by reducing the burden of SASPs.

Studies have shown that senescent cells accumulate in aged human brain organoids [378]. Senolytic therapy not only reduces age‐related inflammation but also resets the transcriptomic aging clock. In postmortem brain samples from individuals infected with SARS‐CoV‐2 or COVID‐19, significantly higher numbers of senescent cells were observed compared with age‐matched controls [381]. Treatment with senolytics was found to inhibit viral replication, prevent neuronal senescence, and improve clinical symptoms in human ACE2‐expressing mouse models, promoting dopaminergic neuron survival and reducing viral and proinflammatory gene expression. These findings suggest that senolytics may serve as a viable therapeutic strategy for combating neurodegenerative diseases—such as AD—triggered by aging and viral infections [378, 382]. In a Phase 1 trial (NCT04063124), D+Q treatment in AD patients showed reductions in senescence‐associated cytokines and chemokines in cerebrospinal fluid, alongside trends of increased Aβ42 levels and elevated IL‐6 and glial fibrillary acidic protein, indicating potential modulation of neuroinflammation and amyloid pathology [382]. Clearance of senescent microglia improved cognitive function in animal models.

In diabetic kidney disease, D+Q reduced the burden of senescent cells in adipose tissue, lowered circulating SASP factors (e.g., IL‐1α, IL‐6, MMP‐9, and MMP‐12), and decreased the number of senescent β‐galactosidase‐positive cells and senescent adipocyte progenitors with limited proliferative capacity. Additionally, senescent cell‐associated macrophage infiltration and crown‐like structures were also reduced (NCT02848131) [383]. These results confirm that senolytic agents can effectively reduce systemic senescence burden in humans. Targeting senescent cells in diabetic retinopathy using BCL‐xL inhibitors may offer durable, disease‐modifying interventions for diabetic macular edema (NCT04537884) [384]. In idiopathic pulmonary fibrosis, D+Q has shown meaningful improvements in patient activity levels, with functional changes correlating with alterations in SASP‐related matrix remodeling proteins, miRNAs, and proinflammatory cytokines [385]. The Phase I trial on the feasibility and tolerance of D+Q in pulmonary fibrosis has been completed at present (NCT02874989) [386] (Table 2).

However, the impact of senolytics on circulating SASP factors remains inconclusive, and their efficacy in treating lung fibrosis itself is still limited [385]. In a study assessing the effects of intermittent D+Q on bone metabolism in postmenopausal women, no significant reduction in overall bone resorption was observed (NCT04313634) [387], highlighting the need for optimized dosing regimens. Moreover, current clinical trials are generally limited by small sample sizes and short observation periods. Given the heterogeneity of senescent cell types across tissues and their differential influence on macrophage regulation, tissue‐specific senolytics are urgently needed. Future research should explore combination therapies, such as senolytics with CAR‐T cells or SASP inhibitors, to enhance efficacy. For example: Combining D+Q with CAR‐T cell therapy could enhance the clearance of senescent cell‐enriched TMEs; D+Q plus SASP inhibitors (e.g., rapamycin) may synergistically eliminate senescent cells while blocking SASP‐driven macrophage activation, thereby enhancing anti‐inflammatory effects; D+Q combined with CSF‐1R inhibitors could more precisely regulate macrophage polarization. In summary, while senolytic therapy holds great promise in targeting age‐related inflammation and fibrosis, ongoing research is essential to refine delivery strategies, expand indications, and develop next‐generation, tissue‐specific senolytic agents.

### 6.3 Macrophage Infusion Therapy

Modern studies have demonstrated that ex vivo expansion and functional modulation of a patient's own macrophages can lead to effective tissue repair and immune regulation upon reinfusion. Autologous macrophage infusion, as a cell‐based therapy, aims to precisely correct complex disease phenotypes through targeted immunomodulation. Several clinical trials have explored the use of autologous macrophages for treating various human cancers. Typically, monocytes (CD14+) are isolated from the patient's peripheral blood via density gradient centrifugation or leukapheresis, then differentiated into macrophages in vitro under controlled conditions using defined cytokine combinations—such as GM‐CSF plus IFN‐γ to induce an M1‐like phenotype or CSF‐1 plus IL‐4 to generate an M2‐like phenotype. These macrophages can subsequently modulate T‐cell subsets (e.g., increasing Treg proportions) and suppress excessive inflammation (e.g., by reducing IL‐6 and TNF‐α levels). However, early trials were conducted before a full understanding of macrophage plasticity and their ability to adapt to different microenvironments was achieved, resulting in limited survival and modest therapeutic efficacy [388].

From animal models to clinical trial design, evidence indicates that macrophages possess strong potential for homing to target tissues and exerting therapeutic effects. In preclinical studies using unmodified macrophages in mice, intravenous or intraorgan delivery showed promising biodistribution patterns. For example, tail vein injection of M1‐ or M2‐polarized macrophages derived from spleen or BM successfully migrated to the kidneys [389]. Notably, these BM‐derived macrophages retained some proliferative capacity, leading to a gradual shift from M2‐like toward M1‐like polarization over time, although the duration of M1/M2 phenotype stability postinfusion remains unclear. Nevertheless, these findings suggest that it may be possible to maintain an M1‐like phenotype through interventions such as iron overload [389]. Early studies relied on intravenous administration or direct transplantation into organs like the lung, liver, or spleen, hoping that endogenous tumor‐derived signals would recruit the cells to the tumor site. However, radiolabeling studies revealed that most infused macrophages initially accumulated in the lungs and were later redistributed to the liver and spleen, with minimal migration to tumors [390]. Although this nontumor tropism fell short of expectations, it opened new avenues for treating lung and liver diseases.

In the context of liver disease, particularly advanced liver fibrosis, macrophages contribute to a persistent inflammatory environment by secreting proinflammatory cytokines, thereby accelerating disease progression [391]. One promising approach is the autologous macrophage‐based phenotypic conversion and collagen degradation system, which leverages M2 macrophages to deliver antifibrotic effects. Upon reinfusion, M2 macrophages secrete anti‐inflammatory cytokines such as IL‐10 and TGF‐β, reduce levels of proinflammatory mediators (e.g., TNF‐α, IL‐6), and promote liver regeneration through the release of hepatocyte GF and VEGF. Importantly, these macrophages can also phagocytose senescent cells, which otherwise drive fibrosis through SASP, thereby interrupting a harmful inflammatory cycle [392].

Preclinical studies have shown that BM‐derived macrophages exhibit superior tissue‐homing ability and regenerative potential. In a Phase 1/2 trial (ISRCTN10368050) of patients with compensated cirrhosis and moderate end‐stage liver disease (MELD score 10–17), autologous MoMF therapy demonstrated a superior safety profile versus standard care [392, 393, 394]. During 360‐day follow‐up, no liver‐related deaths or severe adverse events occurred in the treatment group, whereas the control group experienced four liver‐related SAEs and three deaths. Although no significant differences were observed in biomarkers or health‐related quality of life, exploratory analyses indicated a shift toward an anti‐inflammatory cytokine profile postinfusion. These results support the safety and potential efficacy of macrophage therapy in liver cirrhosis.

Despite its promise, several challenges remain in advancing macrophage‐based therapies for liver disease. Current limitations include suboptimal survival and homing efficiency after intravenous infusion, with macrophages predominantly accumulating in the lungs, liver, and spleen. Optimizing delivery methods—such as local injection or surface modification for enhanced targeting—could improve therapeutic outcomes. Additionally, combining macrophage therapy with senolytic agents (e.g., D+Q) may enhance efficacy by clearing senescent cells that perpetuate inflammation and fibrosis. To address the issue of ECM barriers limiting therapeutic effectiveness, novel delivery systems have been developed. For instance, lipid nanoparticle platforms carrying MFG‐E8 (milk fat globule‐EGF factor 8) can respond to high ROS environments in fibrotic livers, promoting macrophage polarization from M1‐like to M2‐like phenotypes and inducing collagen phagocytosis [391]. This strategy effectively suppresses proinflammatory signaling and facilitates ECM remodeling, offering a promising direction for future liver fibrosis treatments [391].

### 6.4 Genetically Engineered Macrophage‐Based Cell Therapy

Early attempts to use ex vivo cultured antitumor macrophages for adoptive cell therapy revealed significant challenges, particularly regarding phenotypic instability. Upon reinfusion, the therapeutic macrophages often lost their antitumor characteristics due to reprogramming by the tumor or fibrotic microenvironment, sometimes even switching to a disease‐promoting phenotype [395]. In addition, autologous cell therapies are costly and logistically challenging. Therefore, developing genetically engineered, off‐the‐shelf macrophages derived from induced pluripotent stem cells represents a promising strategy to enhance functional stability, reduce costs, and improve clinical feasibility. These cells can be modified using CAR‐M or through metabolic reprogramming to stably adopt either M1‐like or M2‐like phenotypes, thereby enhancing their therapeutic functionality.

In preclinical models of human ovarian cancer xenografts, gene‐modified macrophages exhibited significantly higher cytotoxicity against various tumor cell lines compared with untransduced controls [396]. Similarly, in a murine model of peritoneal metastatic gastric cancer, infusion of myeloid cells engineered to constitutively express IFNβ—a cytokine known to inhibit tumor angiogenesis and upregulate cytotoxic gene expression—markedly suppressed tumor growth, whereas unmodified macrophages accelerated tumor progression [397]. Notably, these IFNβ‐expressing macrophages nearly doubled the median survival time in ovarian cancer‐bearing mice [396], suggesting that genetically enhanced macrophages could serve as a viable treatment option for solid tumors such as gastric cancer and ovarian cancer. Beyond oncology, laboratory‐expanded and genetically engineered alveolar macrophages have shown promise in lung‐targeted therapies. When transplanted into recipient animals, these macrophages specifically home to the lungs, where they fully integrate into the tissue microenvironment and function normally [398]. This feature makes them highly suitable for treating pulmonary diseases such as lung cancer, idiopathic pulmonary fibrosis, and chronic respiratory infections.

A key applications of engineered macrophages is in the treatment of pulmonary alveolar proteinosis, a rare lung disease caused by loss‐of‐function mutations in the GM‐CSF receptor β subunit (Csf2rb), which impair macrophage function and trigger surfactant protein accumulation. Transplantation of wild‐type or gene‐corrected Csf2rb macrophages into the lungs of diseased mice not only significantly improved survival but also demonstrated full integration of the transplanted cells across alveolar and interstitial compartments [399, 400]. This highlights the critical role of the local tissue microenvironment in shaping macrophage identity and function. Moreover, genetic modifications—such as enhancing GM‐CSF or its receptor expression—can further improve macrophage survival and engraftment in the lung and other tissues [399, 400, 401].

With advances in genome editing technologies like CRISPR‐Cas9, it is now feasible to rationally design macrophages with tailored functions for specific diseases. By activating lineage‐specific transcription factors, macrophages can be programmed to migrate to and persist within target organs, enabling more precise and durable therapeutic effects [402]. Strategies to limit macrophage plasticity—such as blocking the NF‐κB signaling pathway—can stabilize tumor‐associated macrophage polarization and enhance antitumor immunity [403]. Additionally, modulating GM‐CSF signaling offers a promising approach to increase macrophage persistence in tissues like the lung [399, 400, 401].

Although genetically engineered macrophages hold great promise for cardiovascular diseases, oncologic, and pulmonary disorders, most evidence remains at the preclinical stage. Future research will focus on optimizing macrophage engineering protocols, improving delivery methods, and ensuring long‐term safety and efficacy. With advancing technological and biological insights, engineered macrophages are emerging as a cornerstone of regenerative medicine and next‐generation immunotherapies.

## 7 Conclusion and Prospects

As an indispensable component of the immune system, macrophages play a dual role in maintaining tissue homeostasis and driving chronic inflammation [19, 23, 24, 25]. Advances in single‐cell technologies have revolutionized our understanding of their developmental origins, tissue‐specific identities, and functional states. It is now well established that many tissue‐resident macrophages originate from embryonic precursors (YS and FL) and are maintained primarily through local self‐renewal rather than continuous replenishment by circulating monocytes, as previously believed [19, 22]. This paradigm shift highlights their remarkable plasticity in response to microenvironmental signals, enabling polarization toward either proinflammatory (M1‐like) or anti‐inflammatory (M2‐like) states [68, 69]. During chronic inflammation, a dynamic immunomodulatory microenvironment is formed through the delicate balance between proinflammatory and anti‐inflammatory cytokines secreted by various immune cells, involving complex metabolic reprogramming and gene regulatory networks. Deciphering these intricate signaling pathways that govern macrophage polarization is essential for elucidating their immunomodulatory functions in both health and disease, offering new insights into their roles in diverse pathological processes ranging from tissue repair to cancer progression.

Emerging technologies such as single‐cell resolution analysis [225, 226] and nanotechnology‐based targeted therapies [391] hold transformative potential for treating diseases mediated by macrophages [32, 224]. These cutting‐edge approaches enable precise modulation of macrophage behavior, opening novel therapeutic avenues for conditions including liver diseases [336, 338], neurodegenerative disorders, and age‐related pathologies [378, 382]. Notably, senolytic therapies—designed to selectively eliminate senescent cells—have shown promising results in alleviating physiological aging and its associated diseases. Preliminary clinical trials suggest that such interventions can reduce the burden of senescent cells and improve symptom outcomes in age‐related conditions such as idiopathic pulmonary fibrosis, diabetic kidney disease, and AD [79, 80, 81, 82, 83, 90, 91, 92, 93]. As research progresses, rigorous evaluation of these innovative therapies in larger and more diverse patient populations will be crucial to establish their long‐term safety, efficacy, and broader applicability, potentially ushering in a new era of macrophage‐centered therapeutic strategies.

Future research should prioritize the investigation of interactions among autophagy, metabolic pathways, and epigenetic regulation in shaping macrophage function, as these mechanisms may reveal novel therapeutic targets. The complexity of macrophage heterogeneity necessitates a deeper understanding of how distinct subpopulations contribute to disease initiation, progression, and clearance in different tissue contexts. Moreover, interdisciplinary collaboration among immunologists, geneticists, bioinformaticians, and clinicians is vital for translating fundamental discoveries into clinical applications. By integrating multiomics data with functional studies, researchers can uncover context‐dependent roles of macrophages in various pathological settings, thereby facilitating the development of more precise and tailored intervention strategies targeting specific disease stages or patient subgroups.

The emergence of personalized medicine approaches guided by individual macrophage profiles and disease contexts represents an exciting frontier in immunotherapy. Leveraging advances in single‐cell analysis and spatial transcriptomics, clinicians may soon be able to stratify patients based on their unique macrophage signatures and microenvironmental cues. Furthermore, developing strategies to modulate macrophage polarization or target specific subpopulations could lead to breakthroughs in treating inflammatory, fibrotic, and degenerative diseases. Addressing the challenges posed by macrophage plasticity and functional diversity will require innovative experimental models and computational tools capable of predicting their behavior in dynamic disease microenvironments. Ultimately, by unraveling the multifaceted roles of macrophages in health and disease, we can pave the way for next‐generation therapies aimed at restoring immune homeostasis and improving outcomes for patients with currently difficult‐to‐treat conditions.

## Author Contributions

Mengyuan Peng led the conceptualization of the topic, wrote the manuscript, and organized the structure. Niannian Li contributed significantly to refining the content and assisting with manuscript preparation. Hongbo Wang contributed significantly to data interpretation and drafting sections of the manuscript. Yaxu Li, Hui Liu, Yanhua Luo, Bao Lang, Weihang Zhang, and Shilong Li also participated in collecting references and refining the content. Liujun Tian and Bin Liu served as corresponding authors and provided critical revisions, guidance, and final approval of the manuscript. All authors have read and approved the final version of the manuscript.

## Ethics Statement

The authors have nothing to report.

## Conflicts of Interest

The authors declare that the study was conducted in the absence of any business or financial relationship that could be interpreted as a potential conflict of interest.

## Data Availability

The authors have nothing to report.
